# SBP‐Box Transcription Factor JcSPL9 Regulates Both Seed Yield and Oil Content in the Biofuel Plant *Jatropha curcas*

**DOI:** 10.1111/pbi.70558

**Published:** 2026-01-23

**Authors:** Mingyong Tang, Xue Bai, Yaoping Xia, Ping Huang, Zeng‐Fu Xu

**Affiliations:** ^1^ CAS Key Laboratory of Tropical Plant Resources and Sustainable Use, Xishuangbanna Tropical Botanical Garden Chinese Academy of Sciences Menglun Yunnan China; ^2^ State Key Laboratory for Conservation and Utilization of Subtropical Agro‐Bioresources, College of Forestry Guangxi University Nanning Guangxi China

**Keywords:** fatty acid composition, inflorescence, *Jatropha*, miR156/*SPL9* module, oil content, seed yield

## Abstract

*Jatropha curcas* is a promising feedstock for biodiesel and bio‐jet fuels production; however, its seed yield is constrained by limited inflorescences. *SPL9* is a member of the SBP‐box gene family that promotes the juvenile‐to‐adult phase transition. Accumulating evidence demonstrated that the miR156/*SPL* module plays important roles in regulating diverse plant developmental processes. Here, we reveal that *JcSPL9* regulates both seed yield and oil content in *Jatropha*. *JcSPL9* is highly expressed in fruits and upregulated with age in *Jatropha*. Overexpression of miR156‐resistant *JcSPL9* (*rJcSPL9*) significantly increased seed yield and oil content, whereas overexpression of *JcmiR156a* had the opposite effects. The highest seed yield in *rJcSPL9* transgenic plants was 80.76% greater than that in the WT plants, with a concomitant 12.6% increase in seed oil content. Correspondingly, *JcmiR156a* transgenic plants displayed 51.67% lower seed yield and 8.28% lower seed oil content compared to WT. Additionally, seed oil fatty acid composition was significantly altered in both *rJcSPL9* and *JcmiR156a* transgenic *Jatropha* and Arabidopsis, as well as in Arabidopsis *spl9* mutants. The key oil biosynthesis genes, including *JcWRI1*, *JcDGAT1*, *JcDGAT2*, and *JcOLEOSIN*, were upregulated in *rJcSPL9* transgenic seeds but downregulated in *JcmiR156a* transformants. This study provides the first evidence that the miR156/*SPL9* module regulates lipid accumulation and fatty acid biosynthesis in seeds. These results highlight *SPL9* as a promising target for enhancing oil yield and quality in *Jatropha* and other oilseed crops.

**Abbreviations**
*AP1*
: *APETALA1*
*AP2*
: *APETALA2*
CaMV: cauliflower mosaic virus
DU: degree of unsaturation
FA: fatty acid
*FT*
: *FLOWERING LOCUS T*
*FUL*
: *FRUITFULL*
*IPA1*
: *IDEAL PLANT ARCHITECTURE 1*
*KRN*
: *KERNEL ROW NUMBER*
LD: long‐day conditions
*LFY*
: *LEAFY*
OS: oxidative stability
qRT‐PCR: quantitative reverse transcription polymerase chain reaction
*SBP*
: *SQUAMOSA PROMOTER BINDING PROTEIN*
*SOC1*
: *SUPPRESSOR OF OVEREXPRESSION OF CONSTANS1*
*SPL*
: *SQUAMOSA PROMOTER BINDING PROTEIN‐LIKE*
*tsh4*
: *tasselsheath4*
UB3: Unbranched3
WAP: week‐after‐pollination
*WFP*
: *WEALTHY FARMER'S PANICLE*

## 1 Introduction

The American Department of Energy predicted that global demand for energy would increase by approximately 35% between 2005 and 2030 (Sun et al. 2017). With the decreasing availability of fossil fuels and the increasing trend of environmental pollution, biodiesel has garnered significant attention as an alternative fuel (Ali et al. 2020; Mardhiah et al. 2017; Mofijur et al. 2016). The use of sustainable biofuels as a source of energy is predicted to be a major contributor to future threats to food security. The use of agricultural plants such as maize, rice, and soybean as feedstocks for large‐scale biofuel production would also conflict with food production, causing food supply shortages, increased food prices, and ethical conflicts (Callegari et al. 2020; Marriott et al. 2016). One potential solution to the food crisis is to produce biofuels from plant species capable of growing on marginal lands (Kazamia and Smith 2014; Sun et al. 2017).

*Jatropha curcas* (hereafter referred to as *Jatropha*), a perennial woody plant species that belongs to the Euphorbiaceae family, is monoecious, with male and female flowers borne on the same inflorescence (Divakara et al. 2010; Pandey et al. 2012; Wu et al. 2011). This species has been propagated as a staple biodiesel and bio‐jet fuels crop because of its multipurpose value, including its high oil content, high biomass productivity, adaptability to marginal lands under a variety of agro‐climatic conditions, and lack of competition with food production (Akashi 2012; Alherbawi et al. 2021; Khalil et al. 2013; Mardhiah et al. 2017; Pandey et al. 2012; Pua et al. 2011). However, the potential of *Jatropha* as a biofuel plant is limited by its low seed production (Akagi et al. 2019). *Jatropha* exhibits an overabundance of vegetative shoots and leaves; thus, a reduction in undesired vegetative growth is imperative (Ghosh et al. 2010; Tjeuw et al. 2015). In addition, poor flowering and branching are important factors that contribute to low seed productivity in crop species (Cui et al. 2014; Divakara et al. 2010). Several strategies have been reported to improve the seed production of *Jatropha*, including promotion of early flowering through *JcFT* overexpression flowering time (Ye et al. 2014), interspecific crossing with *Jatropha integerrima* (Bai et al. 2025), cytokinin‐mediated increases in female flower numbers (Pan et al. 2016; Pan and Xu 2011), and *JcARF19* overexpression to increase seed size (Sun et al. 2017).

In the plant kingdom, microRNA (miR) 156 and miR172 play pivotal regulatory roles in the transition from the vegetative to the reproductive phase (Akagi et al. 2014; Carles and Fletcher 2003; Cheng et al. 2021; Gao et al. 2025). The transcription of miR172 is positively regulated by *SQUAMOSA PROMOTER BINDING PROTEIN‐LIKE 9* (*SPL9*) and *SPL10* (Wu et al. 2009). Moreover, *SPL*s are the direct targets of miR156 (Fornara and Coupland 2009); thus, *SPL* is involved in a miR156 and miR172 signalling cascade (Yu et al. 2015). In *Arabidopsis*, miR156 is highly expressed during early shoot development but decreases as the plants develop, while *SPL9* exhibits an inverse temporal expression pattern (Axtell and Bowman 2008; Wu et al. 2009). Similar patterns were observed in the expression of these two genes in woody species such as *Acacia confusa* , *Acacia colei*, *Eucalyptus globulus* , *Hedera helix* , and *Quercus acutissima* (Wang et al. 2011). Members of the *SPL* family, which represent a class of plant‐specific transcription factors, influence the juvenile‐to‐adult phase transition (Bu et al. 2025; Carles and Fletcher 2003; Hu et al. 2023; Li et al. 2023; Schwab et al. 2005). *SPL* genes have been found in nearly all plant species, including algae and moss. All members of the *SPL* family contain a highly conserved DNA‐binding domain (SBP domain) that is 76 amino acids in length (Wang and Wang 2015).

There are 16 members of the *SPL* family in *Arabidopsis* (Chuck et al. 2007). Overexpression of miR156, which downregulates *AtSPL* genes, prolongs the juvenile phase. Constitutive overexpression of different *AtSPL*s accelerates the juvenile‐to‐adult phase transition (Wang et al. 2009; Wu et al. 2009; Zhao, Liu, et al. 2022), and *AtSPL9*, *AtSP13*, and *AtSPL15* play more important roles in this process than do other *SPL*s (Wang et al. 2021; Xu et al. 2016). *AtSPL9* directly activates the expression of miR172b to facilitate flowering by suppressing the APETALA2 (AP2) protein (Aukerman and Sakai 2003; Wang et al. 2009), which is also involved in seed‐related phenotypes, such as seed mass, yield, and oil biosynthesis (Jofuku et al. 2005). In addition, *AtSPL9* is involved in the regulation of plastochron length and organ size, response to stress, the repression of adventitious root development, anthocyanin biosynthesis, cauline leaf identity, and trichome distribution (Cui et al. 2014; Manuela et al. 2025; Rankenberg et al. 2021; Stief et al. 2014; Wang et al. 2008; Xu et al. 2016; Yu et al. 2010; Zhao, Shi, et al. 2022). Overexpression of *SPL9* can also accelerate flowering in rice ( *Oryza sativa* ) and Chinese cabbage (Luo et al. 2012; Wang et al. 2014). *SPL9* homologues from a basal eudicot tree species ( *Platanus acerifolia* ) can induce early flowering in *Arabidopsis* (Han et al. 2016). In the tree species *Fortunella hindsii* , the miR156‐SPL module regulated the initial phases of somatic embryogenesis induction (Long et al. 2018).

Of the 19 *SPL* genes in the rice genome, *OsSPL14*, which is also known as *ideal plant architecture 1* (*IPA1*) or *WFP* (*WEALTHY FARMER'S PANICLE*), is the most similar to *AtSPL9* (Xie et al. 2006). Many studies have shown that enhanced expression of *OsSPL14* increased panicle branching and grain weight together with increased culm strength (Giaume and Fornara 2021; Jiao et al. 2010; Miura et al. 2010; Wang et al. 2018, 2015; Zhang et al. 2017). And Wang et al. (2018) demonstrated that *OsSPL14* promotes both yield and immunity in rice. In maize, *Unbranched3* (*UB3*), an orthologue of *OsSPL14*, is responsible for the quantitative variation in kernel row number by negatively modulating the size of the inflorescence meristem; moreover, *ub3* mutants present increased kernel row numbers and decreased branching (Du et al. 2017; Liu et al. 2015). Moderate expression of *UB3* suppressed tillering slightly but promoted panicle branching, resulting in increased grain number per panicle in rice (Du et al. 2017; Li, Wang, et al. 2022). Recently, Cao et al. (2021) showed that the wheat *TaSPL14* also regulated spike development and thousand‐grain weight. In soybean, four *GmSPL9* genes redundantly regulate plant architecture (Bao et al. 2019). SPL9 and SPL13 bind to the promoters of *BOP1/BOP2* directly to repress their expression, resulting in delayed establishment of proliferative regions in leaves, which promotes more blade outgrowth and suppresses petiole development (Hu et al. 2023).

*Jatropha* is a perennial plant that has vigorous vegetative growth but relatively poor reproductive growth and low seed yield (Ghosh et al. 2010; Li, Wang, et al. 2022). 15 *JcSPL* genes were identified in *Jatropha*, but only *JcSPL3* was ectopically expressed in *Arabidopsis* (Yu et al. 2020). As mentioned above, *SPL9* in *Arabidopsis* (*AtSPL9*) promotes flowering; and *AtSPL9* orthologues in rice, maize, and wheat are involved in the control of seed yield (Cao et al. 2021; Chuck et al. 2014; Giaume and Fornara 2021; Jiao et al. 2010; Miura et al. 2010; Wu et al. 2009). In the Euphorbiaceae family, *MeSPL9* clustered with *MeSPL15* in the same branch, and overexpression of *rMeSPL9* increased the soluble sugar of cassava (Li, Cheng, et al. 2022). The phylogenetic analysis of the *SPL* family in *Jatropha* exhibited that *JcSPL9* clustered in an individual branch (Yu et al. 2020). Therefore, *Jatropha SPL9* (*JcSPL9*), an orthologue of *AtSPL9*, may also play an important role in promoting reproductive growth in *Jatropha*. In this study, we cloned *JcSPL9* and analysed its roles in growth and development in *Jatropha*. We demonstrate that *JcSPL9* regulates both seed yield and oil content. In particular, we provide the first evidence that miR156/*SPL* module plays a role in regulating fatty acid biosynthesis and lipid accumulation in seeds.

## 2 Materials and Methods

### 2.1 Plant Materials and Growth Conditions

The roots, stems, young and mature leaves, male and female flowers, and fruits of an individual *Jatropha* tree were collected during the summer from the Xishuangbanna Tropical Botanical Garden (XTBG; 21°54′ N, 101°46′ E, 580 m above sea level) of the Chinese Academy of Sciences located in Mengla County, Yunnan Province, Southwest China. Young transgenic *Jatropha* plants were grown in a greenhouse. Mature transgenic *Jatropha* plants were planted in a field plot within the XTBG in 2018–2024. The T1 transgenic *Jatropha* were germinated in August and transferred into the field in November 2020. The cotyledons and young leaves of different‐age *Jatropha* were collected from Kunming, Yunnan Province, China. All tissues prepared for real‐time quantitative reverse transcription polymerase chain reaction (qRT‐PCR) were immediately frozen in liquid N_2_ and stored at −80°C until use. The phenotypes of T0 and T1 *Jatropha* plants were analysed. For each *Jatropha* genotype, more than 25 plants were used for characterisation. The shoot apexes of 5‐month‐old T1 transgenic *Jatropha* plants and the flower buds of 9‐month‐old T1 transgenic *Jatropha* plants were harvested to analyse mRNA transcription levels. *spl9‐4* mutant and *spl9‐4 spl15‐1* double mutant *Arabidopsis* seeds were purchased from the TAIR website (https://www.arabidopsis.org/). The wild type, mutants, and transgenic *Arabidopsis* were germinated on 1/2 MS medium over a one‐week period, after which seedlings were transferred to peat soil in plant growth chambers at 22°C ± 2°C under long‐day (LD) (16 h light/8 h dark) conditions. For each *Arabidopsis* genotype, more than 50 plants were used for collecting seeds.

### 2.2 Cloning of miR156‐Resistant *JcSPL9* ( *rJcSPL9* ) cDNA

Total RNA was extracted from young leaves of *Jatropha* in accordance with the protocol described by Ding et al. (2008). First‐strand cDNA was synthesised using Moloney murine leukemia virus (M‐MLV) reverse transcriptase according to the manufacturer's instructions (TAKARA, Dalian, China). The PCR primers used for cloning of *rJcSPL9* were designed according to the predicted cDNA sequence of *JcSPL9* in *Jatropha* Genome Database (ID: Jcr4S01955.50 or XM_012232354; http://www.kazusa.or.jp/jatropha/) (Sato et al. 2011). All primers used in this research are listed in Table S3. The first round of PCR was amplified via the primers XA39/XA42 and XA40/XA41, which yielded two *rJcSPL9* fragments with mutations. The full‐length sequences of *rJcSPL9* were generated via overlap PCR with the PCR products of the first round by using the primers XA39 and XA40, which introduced *Kpn*I and *Sal*I recognition sites, respectively. *JcmiR156a* was generated via PCR with genomic DNA as template by using the primer XA43 and XA44, which introduced *Kpn*I and *Sal*I recognition sites. The PCR products were subsequently cloned into pGEM‐T vectors (Promega Corporation, Madison, WI, USA) for sequencing.

### 2.3 Construction of Overexpression Binary Vector and Plant Transformation

To construct the plant overexpression binary vector *35S:rJcSPL9* and *35S:JcmiR156a*, the full‐length cDNA sequences of *rJcSPL9* and *JcmiR156a* were excised from the pGEM‐T vector (Promega) using the restriction enzymes *Kpn*I and *Sal*I, and then cloned into a pOCA30 vector containing the cauliflower mosaic virus (CaMV) 35S promoter, respectively. Transformation of *Jatropha* was transformed with *Agrobacterium* strain EHA105 carrying *35S:rJcSPL9* and *35S:JcmiR156a* constructs using the protocol previously described by Pan and Xu (2011) and Fu et al. (2015). Transgenic *Arabidopsis* plants were obtained by *Agrobacterium tumefaciens‐*mediated floral dip (Clough and Bent 1998). Double overexpression lines were produced by genetic crossing. The hybrid lines were obtained by artificial pollination prior to anthesis. Transgenic plants were verified by genomic PCR and RT‐PCR.

### 2.4 Real Time Quantitative Reverse Transcription Polymerase Chain Reaction (qRT‐PCR Analysis

*Jatropha* total RNAs were extracted from frozen tissues as described by Ding et al. (2008). First‐strand cDNA was synthesised from 1 μg of total RNA using the PrimeScript RT Reagent Kit with gDNA Eraser (TAKARA, Dalian, China). The cDNA templates were diluted 5 times with first‐strand cDNA using sterilised double‐distilled water; qRT‐PCR was performed using SYBR Premix Ex Taq II (TAKARA) on a Roche 480 Real‐Time PCR Detection System (Roche Diagnostics, Indianapolis, IN, USA). The primers used for qRT‐PCR are listed in Table S3. qRT‐PCR was performed using three independent biological replicates and three technical replicates per sample. The data were analysed via the 2^−ΔΔCT^ method as described by Livak and Schmittgen (2001). The transcript levels of specific genes were normalised using the *JcActin1* gene (Zhang et al. 2013).

### 2.5 Analysis of Flower, Fruit, and Seed Phenotypes

The flower and fruit anatomy were examined and imaged with a Leica DFC425 camera (Leica, Heerbrugg, Switzerland) equipped with a light Leica DM IRB anatomical lens (Leica). The fruit length and width as well as the seed length, width, and height were measured with an electronic Vernier calliper (to 0.1 mm), and seed weight was measured via an electronic balance. The *Arabidopsis* seed length and width were measured using the analyses function of Leica software.

### 2.6 Analysis of Stem Components

Two‐month‐old T1 transgenic *Jatropha* seedlings growing in the greenhouse were harvested to measure the diameter of their middle stems. The stems were stained with 5% potassium permanganate solution (KMnO_4_) and 5% red ink (Akagi et al. 2018). The stems were then measured for their phloem, xylem, and pith thickness to calculate the areas of the phloem, xylem, and pith, respectively.

### 2.7 Seed Oil Determination by Soxhlet Extraction Method

Seed oil content was measured by AOAC method no. 920.85 (AOAC 1990) with an automatic Soxhlet apparatus (Soxtec 2050, FOSS, Denmark) following the manufacturer's guidelines. The *Jatropha* seed kernels and *Arabidopsis* seeds were ground using a Philips mill and passed through a 16‐mesh sieve. The dried seed powder (0.8–2.5 g) was packed in a thimble and the oil was extracted with petroleum ether (boiling point: 60°C–90°C) for 1.5 h. Upon completion of the oil extraction, the oil was dried at 105°C for 5 h to remove residual water and petroleum ether. The oil content of the samples was calculated on the basis of dry weight of the seeds using the following equation:

$$\text{Oil content}\;\left(\mathrm{mg}/g\right)=\left[\left(W_{b}-W_{a}\right)\times 1000\right]/\text{kernel powder weight}$$

where *W*_*a*_ was the weight of empty flask and *W*_*b*_ refers to weight of flask containing the extracted oil.

### 2.8 Seed Oil Determination by Time‐Domain Nuclear Magnetic Resonance (TD‐NMR)

TD‐NMR determination of seed oil content was carried out with the minispec *mq‐one* Seed Analyser (Bruker Optik GmbH, Ettlingen, Germany), equipped with a sample tube of 40‐mm diameter. For oil determination in *Jatropha* seeds, fresh seeds were dried at 65°C until constant weight (for about 48 h), whereas dry seeds with moisture contents less than 5% were used without drying. A calibration curve was obtained from reference samples of oil extracted by Soxhlet method from mature seeds of *Jatropha*.

### 2.9 Determination of Fatty Acid Composition by Gas Chromatography–Mass Spectrometry (GC–MS)

Approximately 100 μL of *Jatropha* or *Arabidopsis* seed oil was converted to methyl esters with 1 mL of methanol‐sulfuric acid (2.5 M) under 70°Cin a thermostat water bath for 30 min (Khayoon et al. 2012). Then, the crude methyl esters were extracted with 1 mL of hexane before being injected into the GC. The individual fatty acid methyl esters were analysed by an Agilent Technologies 7890A gas chromatograph, equipped with mass spectrometer 5975C (Agilent Technologies, USA). A polar DB‐WAX capillary column (30 m × 0.25 mm i.d. × 0.25 μm film thickness) (Agilent Technologies, USA) was utilised for the separation. The helium carrier gas flow rate was set at 1 mL/min. One microliter of the sample was injected in the split mode at a ratio of 1:30. The oven temperature was initially held at 40°C for 2 min, and then increased to 220°C at a rate of 5°C/min, and finally maintained at 220°C for 10 min (total run time 48 min). The temperatures of the front inlet, transfer line, and ion source were set at 250°C, 250°C, and 230°C, respectively. The MS was taken at 70 eV with a mass range of m/z 35–500. The identification of each fatty acid methyl ester was confirmed by comparing the mass spectra and retention times with those of authentic standards analysed under the same conditions.

The degree of unsaturation (DU) of seed oil and oxidative stability (OS) of biodiesel were calculated according to Feng et al. (2020) by using the following equations:

$$\begin{array}{cc} \mathrm{DU}\;= & \left(\text{monounsaturated}\;\mathrm{Cn}:1,\%\right)+2\times \left(\text{polyunsaturated}\;\mathrm{Cn}:2,\%\right)\\ & +3\times \left(\text{polyunsaturated}\;\mathrm{Cn}:3,\%\right)+4\times \left(\text{polyunsaturated}\;\mathrm{Cn}:4,\%\right);\mathrm{OS}\\ & =-0.0384\times \mathrm{DU}+7.770. \end{array}$$

### 2.10 Determination of Soluble Sugar by High‐Performance Liquid Chromatography

Weigh ~0.5 g of powdered sample into a centrifuge tube, add 4 mL of deionised water, vortex for 30 s and sonicate at room temperature for 30 min. After centrifugation at 5000 r min^−1^ for 5 min, transfer the supernatant to a 5 mL volumetric flask, dilute to volume with water, filter through a 0.22 μm aqueous membrane, and analyse sucrose, D‐fructose, and D‐glucose by high‐performance liquid chromatography.

### 2.11 Analysis of Seed Protein and Starch Contents

The kernel powder was subjected to nitrogen content analysis by the micro‐Kjeldahl method (Bremner 1965), and its protein content was evaluated by multiplying the *N* content by a conversion constant of 5.75 (Mosse 1990). Total starch content was estimated by the anthrone–sulfuric acid method using a spectrophotometer at 630 nm (Hodge and Hofreiter 1962).

## 3 Results

### 3.1 Expression Patterns of *JcSPL9* in *Jatropha*

**FIGURE 1:**
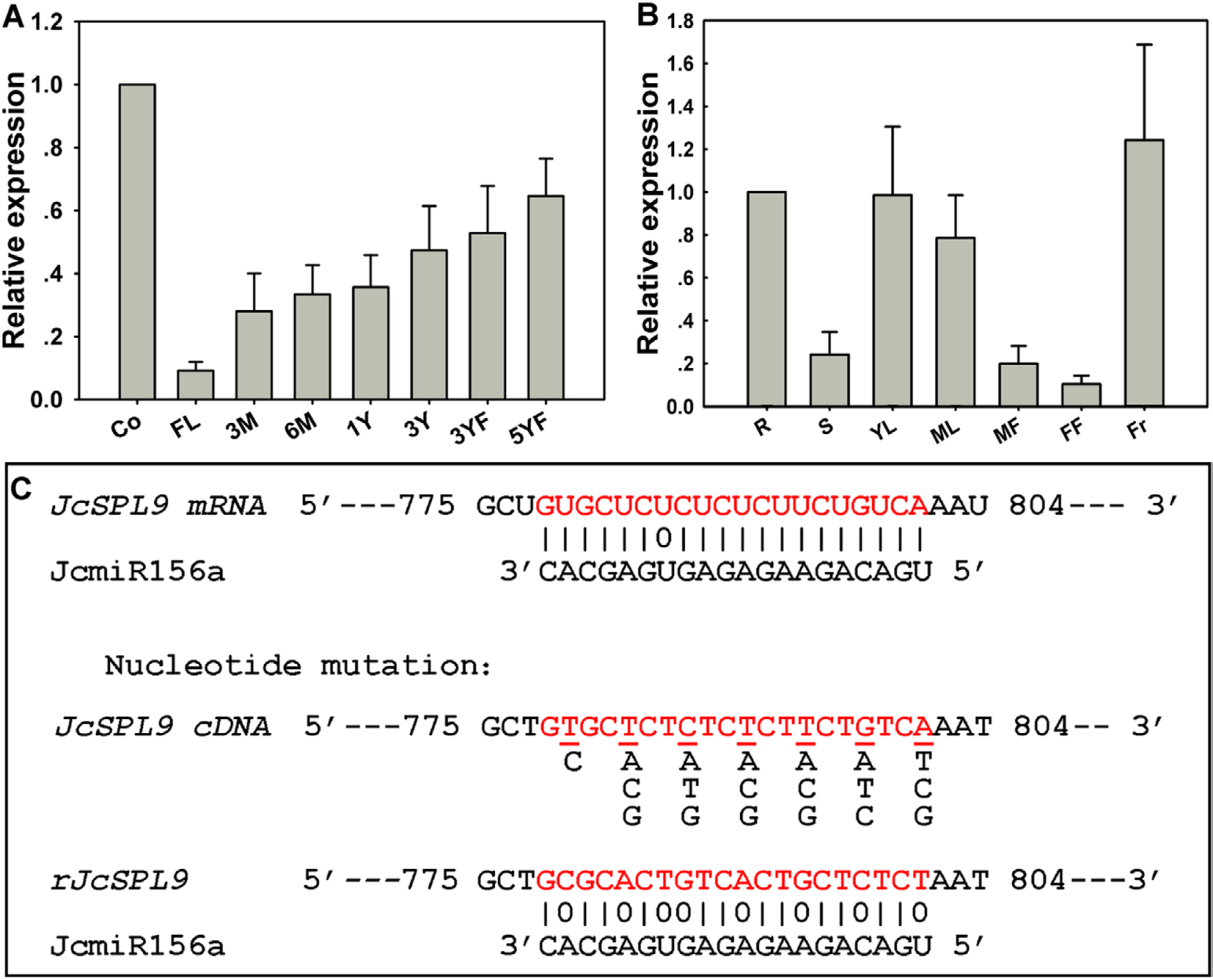
Analysis of the expression patterns of *JcSPL9* in *Jatropha* and prediction of *JcmiR156* target site in *JcSPL9*. (A) Expression levels of *JcSPL9* in cotyledons and young leaves of different ages. Co: Cotyledon, FL: The first leaf of *Jatropha* seedlings, 3 M: Young leaves of 3‐month‐old non‐flowering *Jatropha*, 6 M: Young leaves of 6‐month‐old non‐flowering *Jatropha*, 1Y/3Y: Young leaves of 1/3‐year‐old non‐flowering *Jatropha*, 3YF/5YF: Young leaves of 3/5‐year‐old flowering *Jatropha*. (B) Expression levels of *JcSPL9* in different tissues of a mature *Jatropha* plant. R: Roots, S: Stems, YL: Young leaves, ML: Mature leaves, MF: Male flowers, FF: Female flowers, Fr: Fruits. The qRT‐PCR results were obtained from three independent biological replicates and three technical replicates for each sample; the error bars showed standard deviation. The levels of detected amplicons were normalised using the amplified products of the *JcActin1*. (C) Predicted *JcmiR156* target positions in *JcSPL9* mRNA, the *JcSPL9* transcripts resistant to miR156 (*rJcSPL9*) was generated via mutation of the miR156‐ target site using synonymous codons; *rJcSPL9* sequence showed only 12 nucleotides match with miR156.

*JcSPL9* expression profiles across *Jatropha* development stages were determined by qRT‐PCR. The results show that the expression levels of *JcSPL9* increased with age (Figure 1A). The lowest expression level occurred in the first leaf of the seedlings; as the age increased, the *JcSPL9* expression level increased continuously. The highest expression level occurred in the seedling cotyledons. The second‐highest expression was detected in young leaves of 5‐year‐old flowering *Jatropha* (Figure 1A). *JcSPL9* age‐dependent expression patterns in *Jatropha* were consistent with those reported in other tree species (Wang et al. 2011). We also examined the *JcSPL9* expression levels in different organs of a single *Jatropha* plant. As shown in Figure 1B, *JcSPL9* was expressed in all organs. Expression peaked in fruits, remained high in roots and leaves, and was lowest in stems and female flowers (Figure 1B). These results show that *JcSPL9* expression varied considerably across organs and developmental stages in *Jatropha*, consistent with reports in *Arabidopsis* vegetative tissues (Wang et al. 2009, 2008). In *Arabidopsis*, *SPL9* mRNA was detected in leaf primordia and declined as leaves matured. In the inflorescence apex, *AtSPL9* mRNA was transiently expressed only in the youngest floral primordia (Wang et al. 2009, 2008).

### 3.2 35S: *rJcSPL9* Exhibited a Stronger Phenotype Than 35S: *JcSPL9 Did* in *Arabidopsis*

**FIGURE 2:**
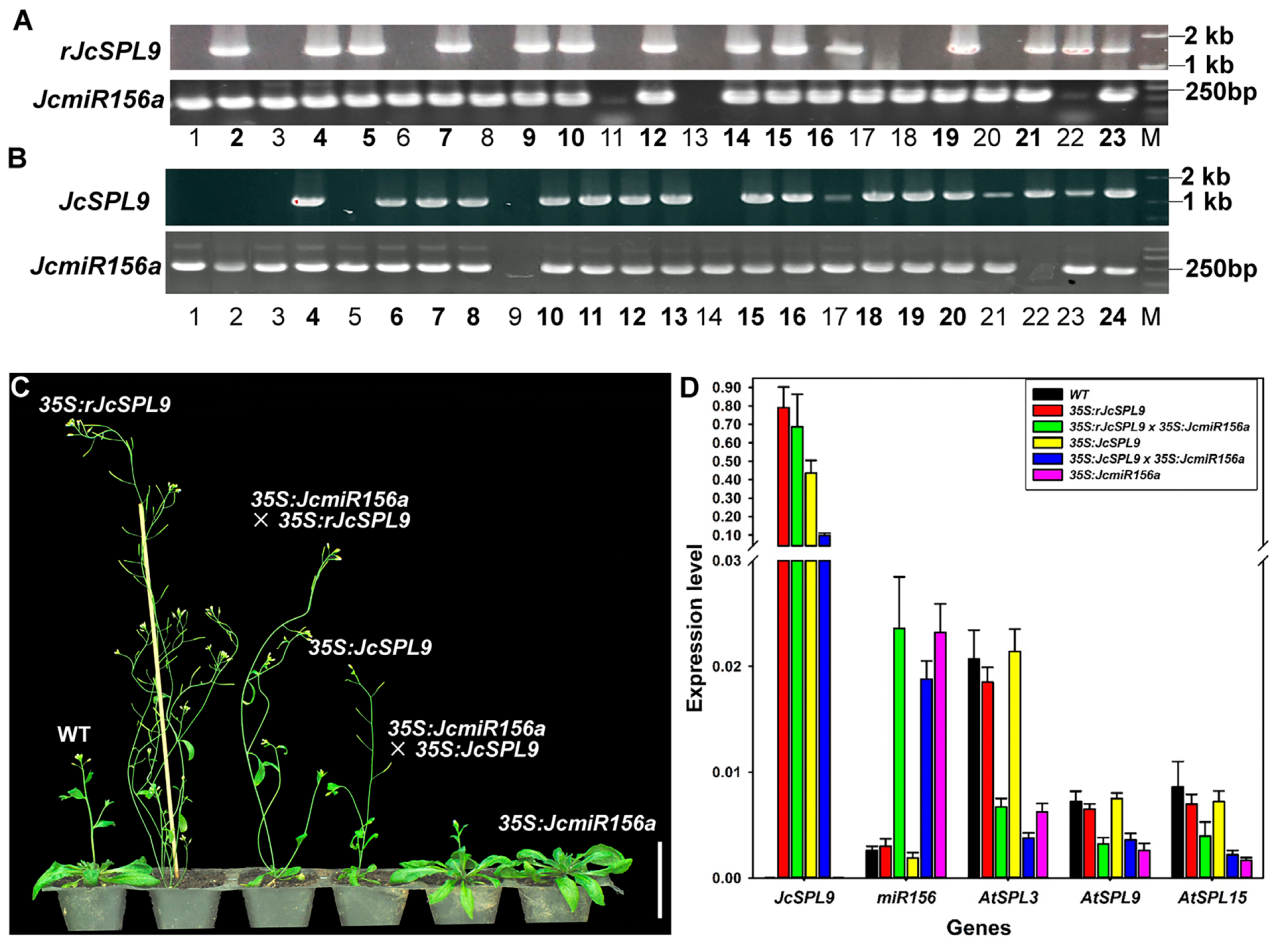
35S:*rJcSPL9* lines flowered earlier than 35S:*JcSPL9* lines in Arabidopsis. (A, B) Identification of *35S:JcmiR156a* × *35S:rJcSPL9/JcSPL9* hybrids through PCR. DNA templates were isolated from artificially pollinated plants. The primer XT126 and XA40 were used to detect *JcSPL9*, and a 1420 bp fragment was amplified. The primer XT126 and XA44 were used to detect *JcmiR156a*, and a 220 bp fragment was amplified. (C) 30‐day‐old *Arabidopsis* of WT, *35S: rJcSPL9, 35S:JcmiR156a* × *35S:rJcSPL9, 35S:JcSPL9, 35S:JcmiR156a* × *35S:JcSPL9*, and *35S:JcmiR156a*. Bar = 5 cm. (D) The relative expression levels of *JcSPL9*, *miR156*, *AtSPL3*, *AtSPL9*, and *AtSPL15* in WT, *35S:rJcSPL9*, *35S:JcmiR156a* × *35S:rJcSPL9*, *35S:JcSPL9, 35S:JcmiR156a* × *35S:JcSPL9*, and *35S:JcmiR156a* plants, *AtActin2* as a reference. The y‐axes represent the relative expression levels of mRNAs to the reference gene. Error bars indicate standard deviations of three biological replicates.

miR156‐resistant *JcSPL9* (*rJcSPL9*) was generated by synonymous codon substitution at the miR156 target site. The *rJcSPL9* sequence showed only 12 nucleotides complementary to*JcmiR156a* (Figure 1C), which was insufficient for miR156 recognition. Transgenic *Arabidopsis* expressing *35S:JcSPL9* and *35S:rJcSPL9* were generated. In this study, 35 lines of *35S:rJcSPL9* transgenic *Arabidopsis* were obtained; 20 (57%) exhibited early flowering. Line L18, showing the strongest phenotype, was selected for flowering time analysis and genetic crosses. Thirty‐two *35S:JcSPL9* lines were obtained; 5 (16%) showed early flowering, with line L11 selected for further analysis. Then the *35S:JcmiR156a* transgenic L7 was used as the female parent to get *35S:JcmiR156a* (L7) *× 35S:rJcSPL9* (L18) and *35S:JcmiR156a* (L7) *× 35S:JcSPL9* (L11) hybrid transgenic plants. Thirteen and fourteen positive F1 plants of two kinds cross combination were identified by PCR, respectively (Figure 2A,B). Subsequently, the flowering time of WT, *35S:rJcSPL9* (L18), *35S:JcmiR156a* (L7) *× 35S:rJcSPL9* (L18), *35S:JcSPL9* (L11), *35S:JcmiR156a* (L7) *× 35S:JcSPL9* (L11), and *35S:JcmiR156a* (L7) was analysed. These results demonstrated that the flowering time of *35S:rJcSPL9* was earlier than *35S:JcSPL9* (Figure 2C, Table S1); after crossing with *35S:JcmiR156a*, the flowering time of *35S:JcmiR156a* (L7) *× 35S:rJcSPL9* (L18) was not obviously changed, and the expression level of *rJcSPL9* was not decreased (Figure 2D). However, the early flowering phenotype of *35S:JcSPL9* (L11) was lost in *35S:JcmiR156a* (L7) *× 35S:JcSPL9* (L11) plants; the expression level of *JcSPL9* was decreased to 25% (Figure 2D). These results indicated miR156‐resistant *JcSPL9* exhibited stronger activity than 35S:*JcSPL9* in *Arabidopsis*, leading us to select *rJcSPL9* for Jatropha transformation.

### 3.3 Overexpression of *rJcSPL9* Promotes Xylem Development and Floral Production in *Jatropha*

In the vegetative stage, *rJcSPL9* overexpression increased axillary bud number and length (Figure S2). Moreover, xylem thickness in transgenic *Jatropha* was 0.15–0.35 mm thicker than that of the WT xylem (Figure S3A,B), and the layers of the xylem thickening expanded significantly in *rJcSPL9* transgenic plants (Figure S3C). These results suggest that *JcSPL9* regulated the stem development by strongly promoting xylem formation and inhibiting phloem and pith development. Furthermore, epidermal wax accumulation was enhanced in *rJcSPL9* transgenic *Jatropha* (Figure S4). During reproductive growth, *35S*:*rJcSPL9* transgenic plants flowered earlier than WT (Figure S5) and exhibited significantly increased female and male flower numbers per inflorescence (Table S2). WT plants produced 8.75 female and 143.63 male flowers per inflorescence. *35S:rJcSPL9* transgenic plants produced 12.83–14.12 female and 181.28–220.75 male flowers per inflorescence. However, the female‐to‐male ratio did not differ between genotypes (Table S2). Compared to the size of reproductive organ, we found inflorescences, male and female flowers, infructescences and fruits were all smaller than WT (Figure 4 and Figure S12).

**FIGURE 3:**
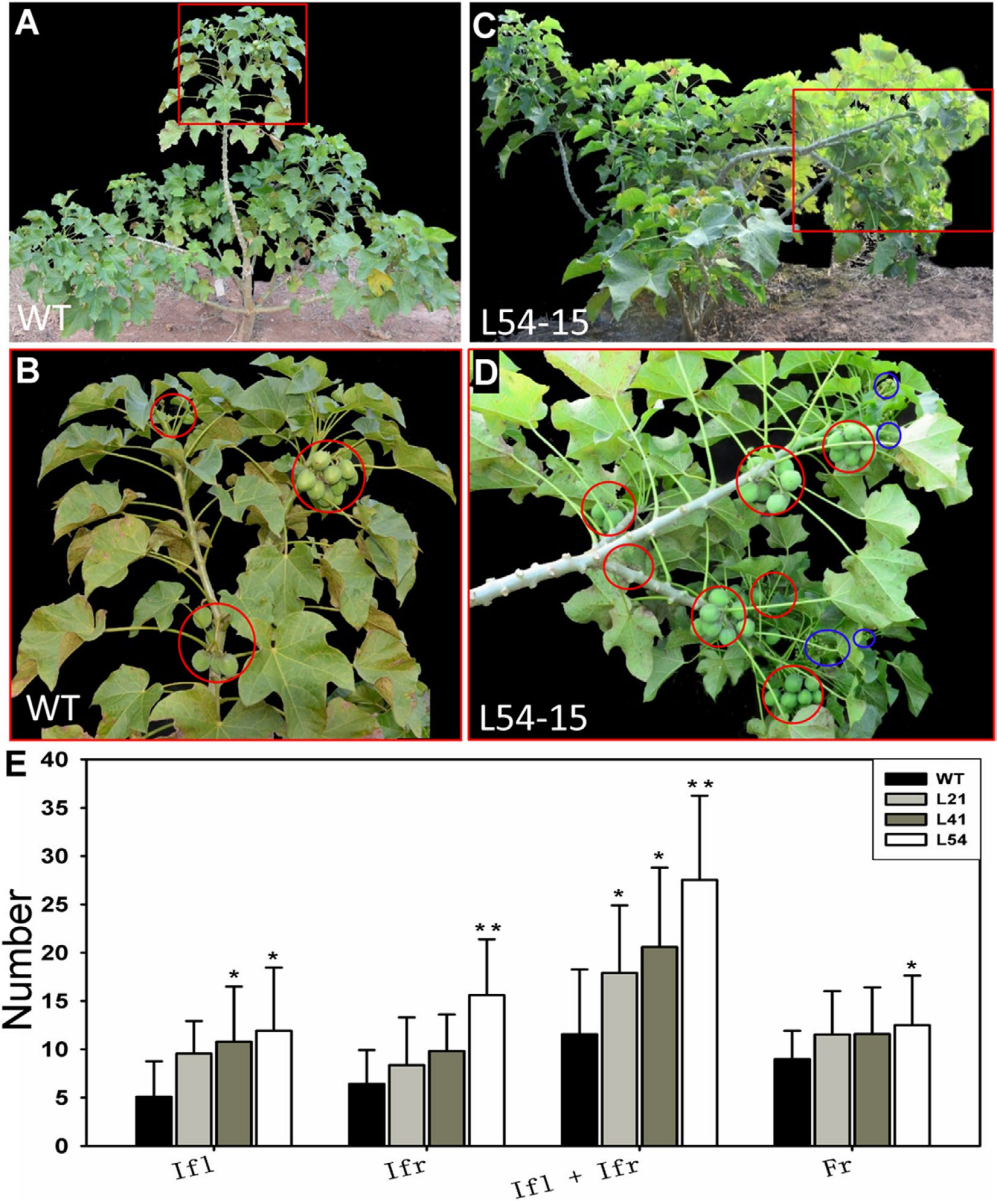
Overexpression of *rJcSPL9* increased the number of inflorescences and infructescences in transgenic *Jatropha*. (A) Ten‐month‐old WT *Jatropha*, with flowers and fruits; (B) high‐magnification image of the boxed portion shown in (A) showing the infructescence, which is indicated by red circles; (C) 10‐month‐old *rJcSPL9* transgenic *Jatropha*, with flowers and fruits; (D) high‐magnification image of the boxed portion shown in (C) showing the inflorescence and infructescence, which are indicated by blue circles and red circles, respectively; (E) Analysis of inflorescence (Ifl) and infructescence (Ifr) numbers per plant and fruit (Fr) number per infructescence. Values are means ± standard deviation (Student's *t*‐test: *, *p* < 0.05; **, *p* < 0.01). The error bars indicate the standard deviations of 20 plants.

Transgenic plants produced significantly more inflorescences and infructescences (Figure 3). Flower number and inflorescence number are two key factors that control seed yield. Therefore, we compared the inflorescence and infructescence numbers on the main stems: 1–3 inflorescences/infructescences were produced on the main stem of the WT plants (Figure 3A,B; Figures S8A and S9A), whereas 5–10 inflorescences/infructescences were produced on the main stem of the transgenic plants (Figure 3C,D; Figures S8B–D and S9B–D). By comparing the inflorescence and infructescence numbers on each plant, both one‐year‐old T0 (Figure S8E) and T1 (Figure 3E) transgenic plants exhibited significantly higher than those of the WT plants. Specifically, WT plants produced ~11 structures per plant in the first year, while T1 transgenic plants produced 18–28 (Figure 3E). As the number of primary branches of the transgenic plants increased, their inflorescence and infructescence numbers were 2 to 3 times greater than those of the WT plants; moreover, compared with that of the WT plants, the fruit number of each infructescence of the L54 plants increased by approximately 3.5 (Figure 3E). The infructescence number of 3‐year‐old WT plants is about 40, whereas the infructescence numbers of 3‐year‐old transgenic plants are more than 70 (Figures S11 and S12A). The increase in flower and inflorescence numbers may be the result of *rJcSPL9* upregulating several genes that affect inflorescence and floral meristem determination, including *JcmiR172*, *JcAP1*, *JcLFY*, and *JcSOC1* (Figure S6C–G). These results indicated that *JcSPL9* was involved in inflorescence meristem and floral meristem determination in *Jatropha*.

### 3.4 Overexpression of *rJcSPL9* Alters Fruit and Seed Morphology

**FIGURE 4:**
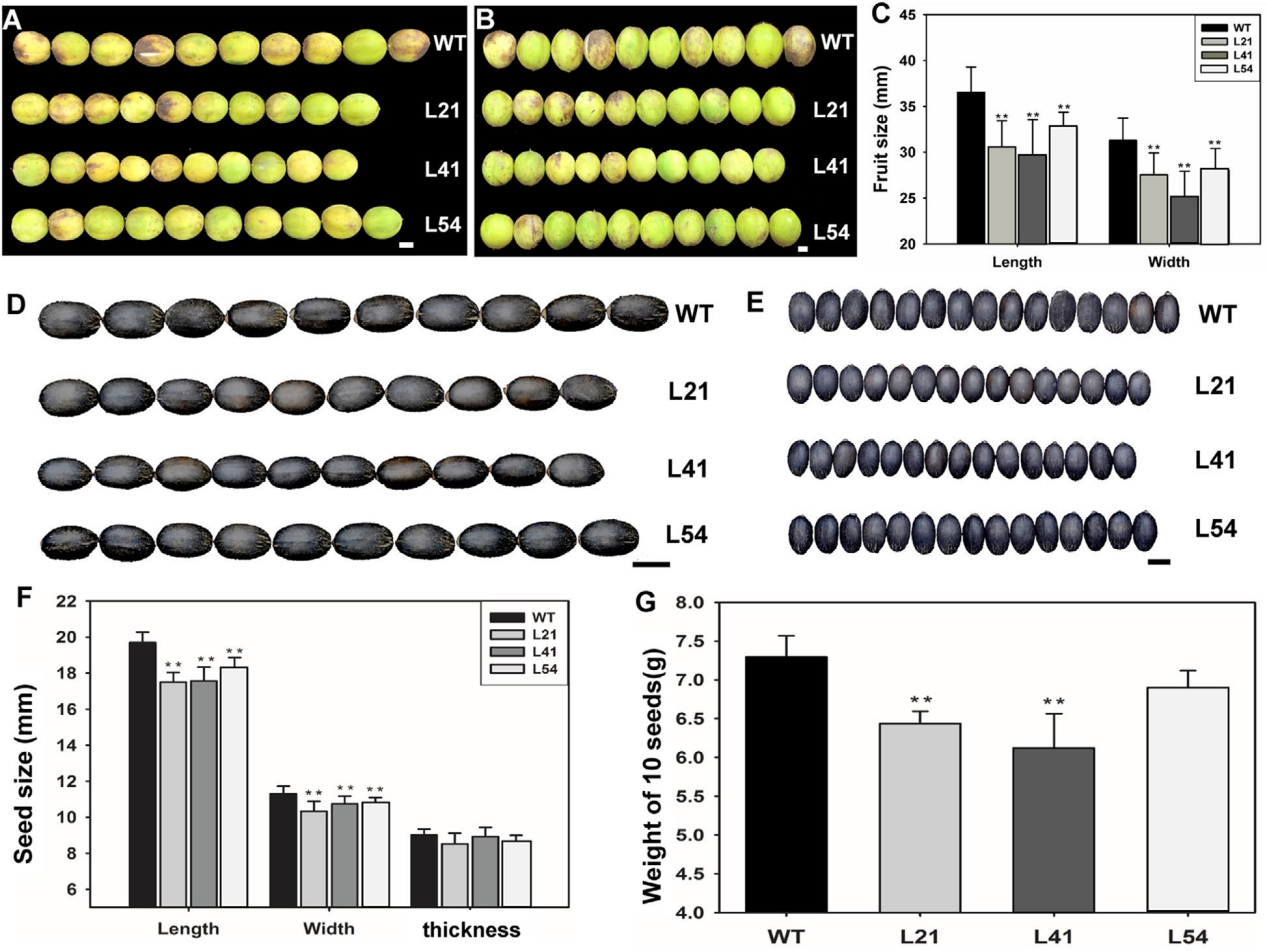
*rJcSPL9* transgenic *Jatropha* produced smaller fruits and seeds. (A, B) Comparison of fruit length (A) and width (B) between WT and *rJcSPL9* transgenic *Jatropha* (L21, L41, and L54). (C) Statistical analysis of fruit length and width in WT and the *rJcSPL9* transgenic *Jatropha*, *N* = 30. (D, E) Comparison of seed length (D) and width (E) between WT and the *rJcSPL9* transgenic *Jatropha*. (F) Statistical analysis of seed length, width, and thickness in WT and the *rJcSPL9* transgenic *Jatropha*, *N* = 30. (G) Statistical analysis of seed weight, *N* = 100. Values are means ± standard deviation (Student's *t*‐test: **, *p* < 0.01). Bars = 1 cm.

**FIGURE 5:**
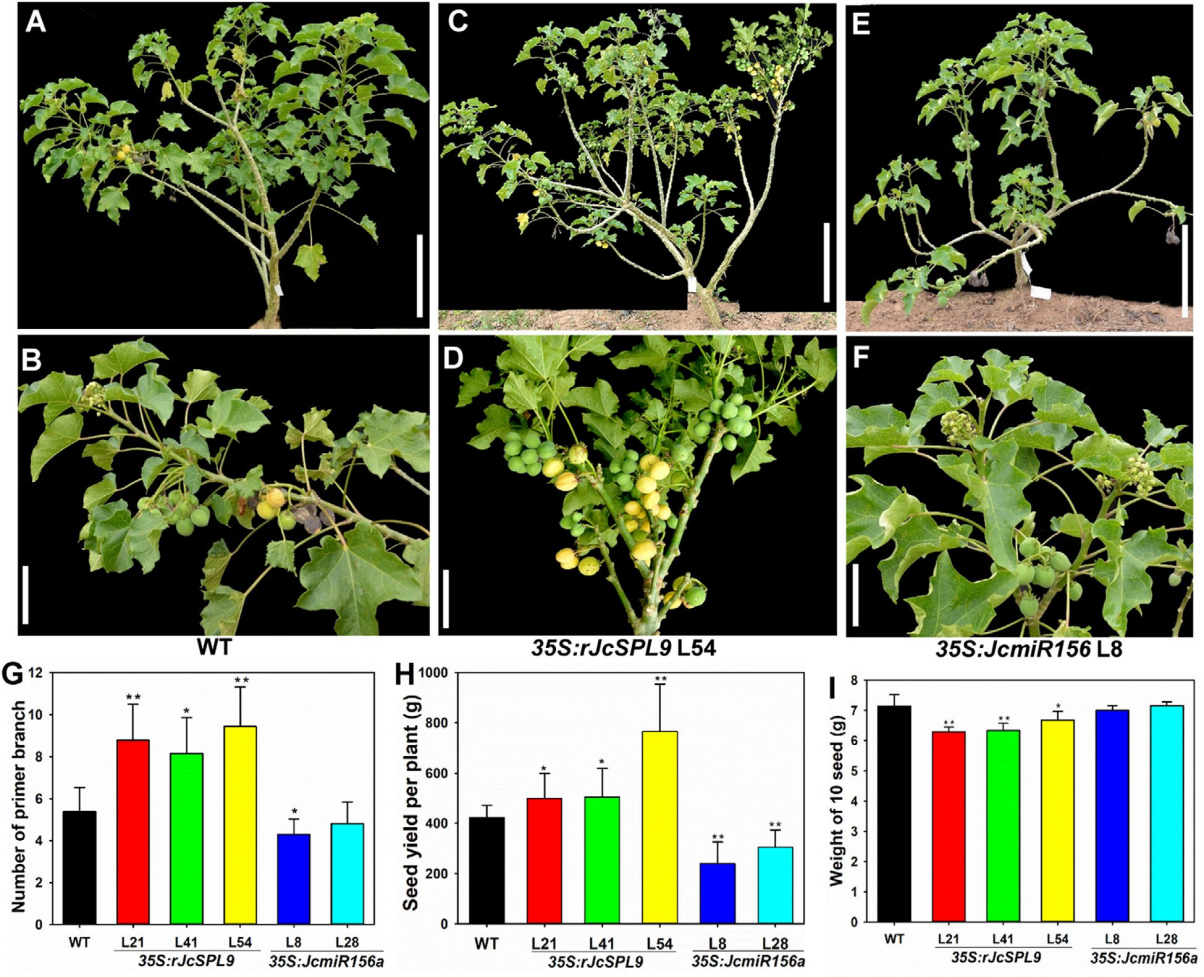
*JcSPL9* positively regulates seed yield in *Jatropha*. (A) WT plant in fruit. (B) A branch of WT in (A). (C) *35S:RrJcSPL9* transgenic plant (L54). (D) A branch of *35S:RrJcSPL9* transgenic plant in (C). (E) *35S:JcmiR156a* transgenic plant (L8). (F) A branch of *35S:JcmiR156a* transgenic plant in (E). (G) Comparison of primary branch numbers in one‐year‐old WT, *35S:RrJcSPL9*, and *35S:JcmiR156a* T1 transgenic *Jatropha*. (H) Comparison of seed yield per plant of WT, *35S:rJcSPL9*, and *35S:JcmiR156a* T1 transgenic *Jatropha*, *N* = 15. (I) Weight of 10 seeds of WT, *35S:rJcSPL9*, and *35S:JcmiR156a* T1 transgenic *Jatropha, N* = 100. All plants were planted via cutting propagation in 2021. Values are means ± standard deviation (Student's *t*‐test: *, *p* < 0.05; **, *p* < 0.01).

Fruit length and width were significantly reduced in *rJcSPL9* transgenic *Jatropha* (Figure 4). L41 plants produced the smallest fruits compared with those of the WT fruit, with length and width reduced by approximately 7 mm (Figure 4C). Transgenic seeds were also smaller than WT seeds (Figure 4D,E). The length and width of the transgenic seeds were significantly decreased (Figure 4F). Moreover, 1‐seed weight was reduced by approximately 0.3–1.0 g in transgenic lines compared to WT (Figure 4G). Specifically, the average weights of 10 seeds were 6.44 g, 6.12 g, 6.90 g, and 7.30 g for L21, L41, L54 and WT, respectively (Figure 4G). Similar results were also found in the following years (Figure 5I). These data indicated that *JcSPL9* played a vital role in the regulation of fruit and seed development in *Jatropha*.

### 3.5 *JcSPL9* Positively Regulates Seed Yield in *Jatropha*

As mentioned previously, *35S:rJcSPL9* transgenic *Jatropha* produced more fruits and smaller seeds than WT did. These traits suggest that *JcSPL9* substantially impacts Jatropha agronomic performance. To evaluate seed yield, 100 T1 transgenic plants were field‐grown and first‐year seed production was compared with WT. Per‐plant seed weight analysis revealed first‐year average yields of 181.13 g, 199.38 g, and 235.71 g for L21, L41, and L54, respectively, compared to 130.40 g for WT (Figure S10A); the seed yield of transgenic line L54 was 80.76% greater than that of WT. To confirm the results, seed yield was monitored over the following 2 years. In the third year, transgenic plants also generated more infructescence and achieved higher seed yield (Figure S12A). The average seed yield of each transgenic plant were 914.36 g, 914.36 g, and 1128.34 g for L21, L41, and L54, respectively, compared to 708.25 g for WT (Figures S11 and S12A); Line L54 exhibited a 59.31% yield increase over WT.

Additionally, WT, *35S*:*rJcSPL9*, and *35S*:*JcmiR156a* T1 plants were propagated by cuttings in 2021 for seed yield analysis in 2022. Results showed that the average seed yield were 499.29 g, 505.45 g, and 765.23 g for L21, L41, and L54, respectively, whereas the average seed yield of each WT plant was only 422.65 g. The average seed yield of *35S:JcmiR156a* T1 transgenic plants was 241.04 g for L8, and 305.62 g for L28 (Figure 5A–G). Notably, L54 exhibited an 81.06% increase in seed yield over WT, while 35S:JcmiR156a line L8 showed a 51.67% decrease. *JcSPL9* expression in *35S:JcmiR156a* transgenic plants was reduced to 25% of WT levels (Figure S6A). These results indicate that *JcSPL9* positively regulates seed yield in *Jatropha*.

### 3.6 *JcSPL9* Regulates Oil Content and Fatty Acid (FA) Composition

**FIGURE 6:**
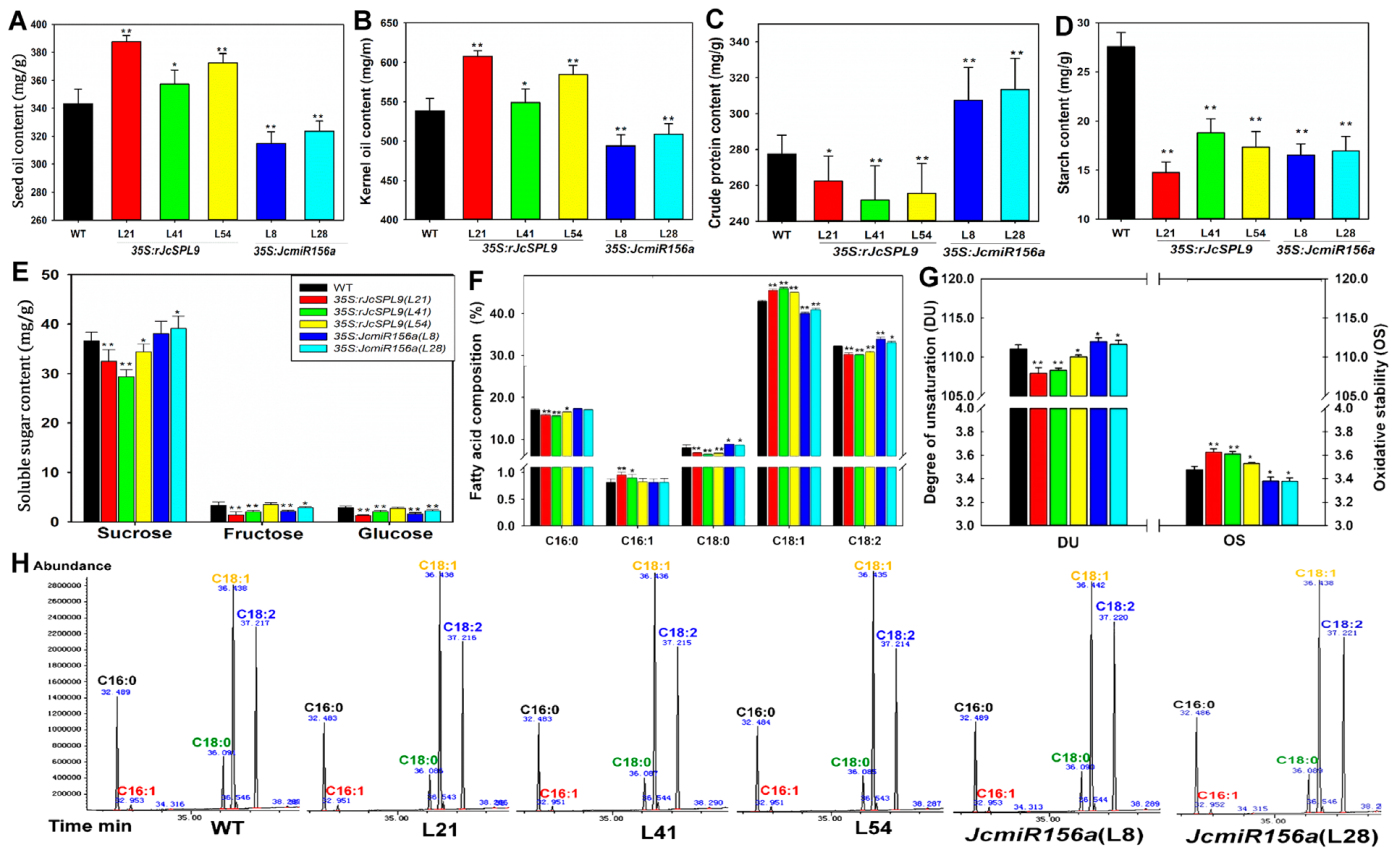
Comparison of the oil, protein, starch, soluble sugar content, and fatty acid composition of seeds from WT, *35S:rJcSPL9*, and *35S:JcmiR156a* T1 transgenic *Jatropha*. (A, B) The seed oil content (A), and kernel oil content (B) among WT, *rJcSPL9* (L21, L41, and L54), and *35S:JcmiR156a* T1 transgenic *Jatropha*. (C–E) The crude protein (C), starch (D), and soluble sugar contents (E) in WT, *rJcSPL9* T1 transgenic *Jatropha* and *JcmiR156a* T1 transgenic seeds. (F) Comparison of the kernel fatty acid composition of WT, *rJcSPL9*, and *JcmiR156a* transgenic *Jatropha*. (G) Comparison of the degree of unsaturation (DU) and oxidative stability (OS) of seed oil from WT, *rJcSPL9*, and *JcmiR156a* transgenic *Jatropha*. (H) Fatty acid composition of seeds by GC–MS. The plants used for this experiment were planted by cutting propagation in 2021. Values are means ± standard deviations, which were calculated from at least 100 seeds independently collected from three independent WT, *rJcSPL9*, and *JcmiR156a* transgenic plants. Asterisks denote significance compared with WT plants (Student's *t*‐test: *, *p* < 0.05; **, *p* < 0.01).

When the oil content of dry seeds of T1 plants was measured, the results showed that the oil content of transgenic seeds was 346.4 mg/g, 315.1 mg/g, and 318.5 mg/g for L21, L41, and L54, respectively, while the oil content of the WT seeds was approximately 307.7 mg/g (Figure S10B). The highest oil content was observed in L21, representing an increase of approximately 39.1 mg/g (12.63% relative increase). However, kernel percentage did not differ significantly among genotypes (Figure S10C). Oil content in transgenic kernels increased by 10.4 mg/g to 37.1 mg/g (2.14% to 7.76% relative increase). Specifically, kernel oil content was 518.0 mg/g, 493.9 mg/g, 491.8 mg/g, and 481.8 mg/g for L21, L41, L54, and WT, respectively (Figure S10D). The highest oil content was observed in L21 kernels, representing an increase of 36.2 mg/g (7.54% relative increase). Based on these data, oil production per plant was 40.12 g, 62.74 g, 62.83 g, and 75.07 g for WT, L21, L41, and L54 in the first year, respectively, representing increases to 1.564, 1.566, and 1.871‐fold for L21, L41, and L54, respectively. Furthermore, seed oil content in rJcSPL9 line L21 increased by 36.0 mg/g (11.11% relative increase) and 41.2 mg/g increase (12.01% relative increase) in the second and third years, respectively, despite slight decreasing in seed weight (Figure S12C, Figure 6A). To confirm that *JcSPL9* regulates oil content, we determined the oil content in T1 *35S:JcmiR156a* transgenic *Jatropha* plants. The results showed that seed oil content of *35S:JcmiR156a* lines L8 and L28 was 314.8 mg/g and 325.2 mg/g, (Figure 6A), which exhibited an 8.28% and 5.22% relative decrease, respectively. The kernel oil content was also significantly decreased in *35S:JcmiR156a* plants (Figure 6B). We also analysed the contents of crude protein, starch, and soluble sugars; these results showed that the contents of these components were almost decreased in *rJcSPL9* transgenic plants but increased in *JcmiR156a* transgenic plants (Figure 6C–E).

To further confirm the function of *SPL9* in the regulation of oil accumulation in *Arabidopsis*, we compared the oil content in WT, *spl9* mutant, and *35S:JcmiR156a* transgenic *Arabidopsis*. These results exhibited that the oil contents were 392.5 mg/g, 344.0 mg/g, 350.5 mg/g, 373.0 mg/g, and 378.2 mg/g in WT, *spl9* mutant, *spl9 spl15* double mutant and *35S:JcmiR156a* transgenic *Arabidopsis* L2 and L7, respectively (Figure S15C). The oil content in *spl9 mutant*, *spl9 spl15* double mutant, and *35S:JcmiR156a* transgenic *Arabidopsis* significantly decreased, with an approximately 48.5 mg/g (12.36% relative) decrease in *spl9* mutant, a 42.0 mg/g (10.70% relative) decrease in *spl9 spl15* double mutant and a 19.5 mg/g and 14.3 mg/g (4.97% and 3.65% relative) decrease in *35S:JcmiR156a* transgenic *Arabidopsis*. Overexpression of *rJcSPL9* in WT and *spl9‐4* mutant plants also increased seed oil content (Figure S15D). These results indicate that, in addition to regulating seed yield, *JcSPL9* also regulates lipid accumulation in seeds.

Fatty acid (FA) composition of oil from *rJcSPL9* and *JcmiR156a* transgenic seeds was detected by GC–MS. These results exhibited that the C16:0 mol% decreased significantly by 3.70%–9.25% in *rJcSPL9* transgenic seed*s*; the C18:0 decreased significantly by 15.01%–20.45% in *rJcSPL9* transgenic seeds but increased by 3.52%–4.21% in *JcmiR156a* transgenic seeds. C18:1 increased significantly by 3.54%–7.04% in *rJcSPL9* seeds but decreased significantly by 5.02%–5.45% in *JcmiR156a* transgenic seeds. C18:2 decreased by 4.08%–5.75% in *rJcSPL9* transgenic seeds, whereas it increased by 4.73%–5.81% in *JcmiR156a* transgenic seeds (Figure 6F). These FA composition changes significantly reduced the degree of unsaturation (DU) in *rJcSPL9* transgenic seed oil, potentially improving biodiesel oxidative stability (OS) compared to WT, while the DU and OS of the *JcmiR156a* transgenic seeds were opposite (Figure 6H).

To verify the role of *SPL9* in regulating FA composition, individual FAs were also determined in oil extracted from *Arabidopsis* seeds of wild‐type, *spl9* mutant, 35S:*JcmiR156a* transgenic WT and *spl9* mutant *Arabidopsis*. These results showed that C18:0, C18:2, and C20:1 mol% were significantly increased in *spl9* mutant, *spl9/spl15* double mutant, and *JcmiR156a* transgenic *Arabidopsis*, whereas both C18:1 and C18:3 mol% were significantly decreased in the mutants and *JcmiR156a* transgenic *Arabidopsis* (Figure S15E). Additionally, in contrast to the *spl9* mutant, the *spl9/spl15* double mutant showed significant changes in C16:0 mol% (Figure S15E). These results indicate that miR156 and its target *SPL9* are involved in regulating FA biosynthesis in both *Arabidopsis* and *Jatropha*.

### 3.7 *JcSPL9* Upregulates the expression of Oil Biosynthesis Genes

**FIGURE 7:**
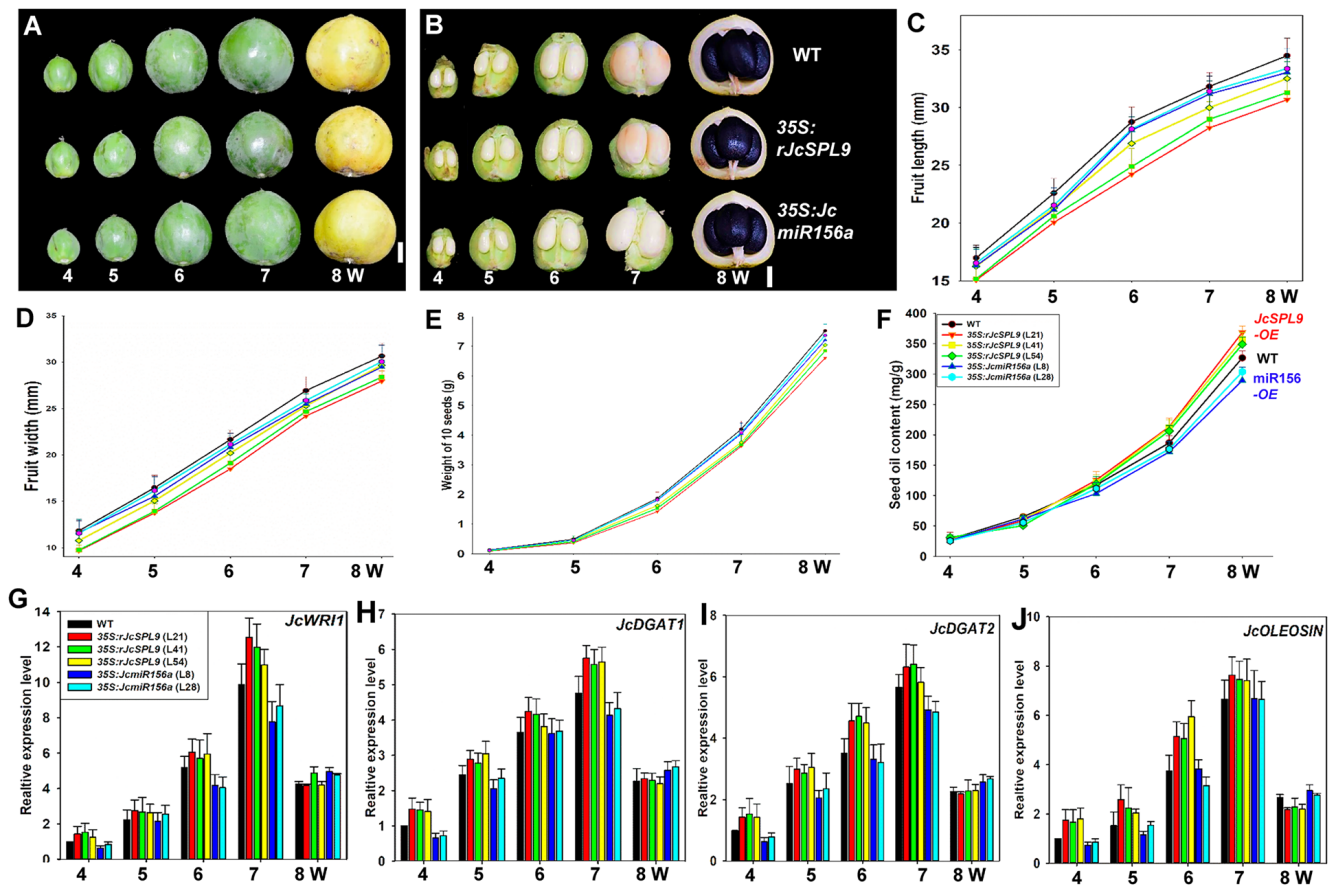
Comparison of oil content and oil biosynthesis gene expression at different stages. (A) 4‐ to 8‐week‐old fruits from WT and transgenic *Jatropha* lines *35S:RrJcSPL9* (L21) and *35S:JcmiR156a*(L8), bar = 1 cm. (B) 4‐ to 8‐week‐old fruits and seed from WT, *35S:rJcSPL9* (L21), and *35S:JcmiR156a* (L8) T1 transgenic *Jatropha*, bar = 1 cm. (C, D) Statistic analyse the fruit length (C) and fruit width (D) from WT, *35S:RrJcSPL9*, and *35S:JcmiR156a* T1 transgenic *Jatropha* from 4 weeks to 8 weeks. (E) Statistic analyzes the weight of 10 seeds of WT, *35S:RrJcSPL9*, and *35S:JcmiR156a* T1 transgenic *Jatropha* from 4 weeks to 8 weeks. (F) The seed oil content of WT, *35S:RrJcSPL9*, and *35S:JcmiR156a* T1 transgenic *Jatropha* from 4 weeks to 8 weeks. (G‐J) Oil biosynthesis gene expression levels, including those of *JcWRI1* (G), *JcDGAT1* (H), *JcDGAT2* (I), and *JcOLEOSIN* (J), in seeds at different develop stages in WT and *transgenic* plants. Values are means ± standard deviations, which were calculated from at least 15 fruits or 40 seeds from each line.

Fruit and seed morphology as well as oil content were analysed at different developmental stages. These results showed that seed weight and oil content increased slowly during early stages, especially before 5 week‐after‐pollination (WAP), but rapidly during late stages, especially the stages 7–8 WAP (Figure 7E,F). Expression of oil biosynthesis genes in the seed at the 4–8 WAP was further detected by qRT‐PCR. During seed development, expression levels of *JcWRI1*, *JcDGAT1*, *JcDGAT2*, and *JcOLEOSIN* were lower at 4 WAP, then increased at 6 WAP, and peaked at 6–7 WAP (Figure 7G–J). Combined with these results, the stage with higher oil‐related gene expression corresponded to the key oil biosynthesis stage (Figure 7F).

In this study, oil content increased in *rJcSPL9* transgenic seeds and decreased in *35S:miR156* transgenic seeds compared to WT *Jatropha*. When comparing across genotypes and developmental stages, differences in seed weight and oil content occurred at 6–8 weeks after pollination and persisted until maturity (Figure 7F). Differential expression of oil biosynthesis genes was observed at 4–7 week‐after‐pollination (Figure 7G–J); gene expression divergence occurred slightly earlier than did oil content divergence. Comparison of oil biosynthesis gene expression revealed that the expression abundances of *JcWRI1*, *JcDGAT1*, *JcDGAT2*, and *JcOLEOSIN* were all increased in the *rJcSPL9* transgenic seeds but decreased in the *35S:JcmiR156a* transgenic *Jatropha* seeds (Figure 7G–J).

## 4 Discussion

### 4.1 *JcSPL9* Positively Regulates Seed Yield in *Jatropha*

Plant branching patterns are crucial for light interception efficiency and resource adaptation. *35S*:*rJcSPL9* transgenic plants developed more axillary buds at the young seedling stage and more branches at the mature stage (Figure S2, Figure 5). *35S*:*rJcSPL9* transgenic *Jatropha* produced more branches, inflorescences, and infructescences than WT (Figure 3, Figures S8, S9, S11, S12A). In this study, *35S*:*rJcSPL9* transgenic plants developed more axillary buds at the young seedling stage and more branches at the mature stage (Figure S2, Figure 5). The *SPL* module's conserved function in tillering/branching has been confirmed in many plant species. In rice, *OsSPL14* (the *AtSPL9* homologue) negatively regulates axillary bud outgrowth while positively regulates panicle branch number by enhancing meristematic activity and cell proliferation (Jiao et al. 2010; Luo et al. 2012). High *OsSPL14* expression increases primary and secondary branches (Miura et al. 2010). Effects of other *SPL* genes (such as *SPL7/13/16/17*) on tillering and panicle branching have also been observed in rice (Si et al. 2016; Wang et al. 2015, 2012). In *Arabidopsis*, SPL9 physically interacts with DELLA proteins (Wang et al. 2019; Yu et al. 2012). In *Jatropha*, overexpression of *rJcSPL9* promoted axillary bud outgrowth and branching (Figure 1), consistent with previous reports that GA promoted shoot branching (Ni et al. 2015, 2017). We propose that GA promotes branching in *Jatroph*a might by regulating DELLA‐JcSPL9 interactions. This notion is supported by a recent study showing that GA represses *Arabidopsis* axillary bud formation by modulation of DELLA‐SPL9 complex activity (Zhang et al. 2020). However, further work is needed to elucidate how GAs modulate shoot branching via opposing pathways in *Jatropha* and *Arabidopsis*. Thus, increased branching indirectly improved the seed yield in rice and *Jatropha* (Figure 5H, Figure S10).

Increased inflorescence and flower numbers, combined with earlier flowering time (Figure S5, Table S2), led to significantly more infructescence (Figure 3E, Figures S8E and S12A). Although the fruit and seed sizes of transgenic plants were smaller, all *35S*:*rJcSPL9* transgenic *Jatropha* plants produced significantly higher seed yield, and the seed yield of transgenic line L54 plants was 80.76% greater than that of the WT plants (Figure 5H, Figures S10A and S12A). On the contrary, the seed yield of *JcSPL9* decreased plants generated by overexpressing miR156 was significantly reduced (Figure 5G). The expression of flowering‐related genes, including *JcLFY*, *JcSOC1*, *JcAP1*, *JcFUL*, and *JcmiR172*, was upregulated in *rJcSPL9* transgenic plants (Figure S6). Therefore, we concluded that *JcSPL9* activated flower identity genes and directly promoted reproductive growth, which increased the inflorescence and flower numbers and further led to an increase in the seed yield.

Studies reporting that *SPL* genes positively regulate seed yield have been documented in rice and tomato (Cui et al. 2020; Wang, Yu, et al. 2017). Both the grain weight and yield increased in lines overexpressing *OsSPL13*, *OsSPL14*, or *OsSPL16* (Miura et al. 2010; Si et al. 2016; Wang and Wang 2015; Wang et al. 2015, 2012). Moreover, overexpression of *OsSPL14* increased the thousand‐grain weight from 27.2 g in control plants to 30.2 g in transgenic plants (Jiao et al. 2010). Moreover, the varieties in which *OsSPL14* was upregulated presented a 10% increase in grain yield in test plots (Jiao et al. 2010). In tomato, both miR156‐overexpression lines and *SlSPL13*‐RNAi plants exhibited reduced numbers of flowers and fruits, leading to a significant decrease in yield per plant (Cui et al. 2020). In maize, *UB3* is an orthologue of *AtSPL9* and is also homologous to *UB2* and *TSH4*. Double mutants of *ub2* and *ub3* displayed reduced kernel row numbers in maize (Chuck et al. 2014; Du et al. 2017; Liu et al. 2015). The loss‐of‐function phenotypes of *ub2/ub3/tsh4* mutants in maize are associated with reduced yields (Chuck et al. 2014), which are opposite to the phenotypes of two dominant gain‐of‐function alleles in rice, *OsSPL14* ^ipa1^ and *OsSPL14* ^WFP^, which improve grain yield by increasing panicle branching (Jiao et al. 2010; Miura et al. 2010).

The seed yield of all *35S*:*rJcSPL9* transgenic lines were significantly increased, with the line L54 producing the highest yield (Figure 5G, Figures S10A and S12A). However, line L54 exhibited the lowest *JcSPL9* expression level (Figure S6A). The seed yield of *35S*:*JcmiR156a* transgenic *Jatropha* were 51.67% less than that of WT (Figure 5G), and the expression level of *JcSPL9* was reduced to a quarter of WT in *35S:JcmiR156a transgenic* plants (Figure S6). Similarly, Wang et al. (2015) reported that moderate *SPL* gene expression may constitute a strategy for increasing rice yield in breeding. Weaker expression of *OsSPL14* leads to the optimal combination of tiller number and panicle size as well as increased grain yields in rice (Zhang et al. 2017). Moreover, both tiller and panicle branching are greatly reduced by overexpressing all *OsSPL7/14/16/17*, indicating that *SPL*s promote panicle branching only at optimal levels (Wang, Qiao, et al. 2017). This conclusion is further strengthened by maize *UB3*, which enhances rice panicle branching in moderate‐overexpression lines, but the opposite effect was observed in high‐overexpression lines (Du et al. 2017). Therefore, *SPL* expression must be fine‐tuned to favourable levels to increase productivity. In addition, *SPL* genes can also control fruit ripening and fruit yield in tomato and kernel row number in maize in a dosage‐dependent manner (Cui et al. 2020; Liu et al. 2015; Wang and Wang 2015), suggesting that modifying *SPL* expression offers a feasible strategy for crop improvement. Furthermore, recent studies have shown that *OsSPL14* promotes both yield and immunity in rice (Wang et al. 2018).

### 4.2 Regulation of Oil Content and Fatty Acid Composition by *JcSPL9*

Several RNA‐seq and bioinformatic studies have predicted that miR156 and its target *SPL* genes might be involved in fatty acid and lipid metabolism in seeds of several species, including *Brassica napus* (Picq et al. 2014; Wang et al. 2016; Wang, Qiao, et al. 2017), oil palm (Zheng et al. 2019), and tree peony (Yin et al. 2018). In this study, for the first time, we demonstrate that the seed oil content and fatty acid composition were significantly altered in *rJcSPL9* transgenic *Jatropha* and *spl9* mutants of *Arabidopsis*, and in *JcmiR156a‐*overexpressing *Jatropha* and *Arabidopsis* (Figures 6, 7 and Figures S13 and S15). The seed oil content in both *Jatropha* and *Arabidopsis* was positively regulated by *SPL9* (Figure 6A and Figure S15C,D). There was no significant difference in oil content of seeds collected before 6 WAP in WT, *rJcSPL9* and *JcmiR156a* overexpressing plants (Figure 7). After 6 WAP, the seed oil content was obviously increased in *rJcSPL9* transgenic *Jatropha* and decreased in *JcmiR156a* transgenic *Jatropha* (Figure 7). Ultimately, transgenic plants exhibited a 112.63% increase in relative seed oil content, corresponding to an absolute increase of 39.1 mg/g. In the first year, oil production per plant increased by 35 g (1.87‐fold) and further rose to 134 g (1.55‐fold) in the second year. Correspondingly, the expression levels of *JcWRI1, JcDGAT1, JcDGAT2*, and *JcOLEOSIN* were increased in *rJcSPL9* transgenic *Jatropha* and decreased in *JcmiR156a* transgenic plants; especially in seeds at 5–7 WAP (Figure 7F). These results indicate that *JcSPL9* may regulate lipid accumulation through activating expression of *JcWRI1, JcDGAT1, JcDGAT2*, and *JcOLEOSIN* in seeds.

At the metabolic level, increased oil accumulation in the *rJcSPL9* transgenic plants was accompanied by significant reductions in crude protein and starch contents in the seed kernels. Specifically, crude protein content decreased by approximately 25 mg/g DW in the most affected line (Figure 6C), while starch content decreased by about 13 mg/g DW (Figure 6D). Soluble sugar content also showed a slight decline (Figure 6E). The decreases in crude protein, starch, and soluble sugar contents closely matched the 41.2 mg/g increase in oil content. The comparative transcriptome analysis revealed that in the transgenic kernels 5–7 weeks after pollination, the expression levels of genes related to lipid synthesis, protein metabolism, and glycolysis had undergone significant changes in most cases (Figure S14). We propose that *JcSPL9* regulates carbon flux allocation between metabolic pathways, thereby coordinating oil biosynthesis and promoting optimal carbon partitioning during *Jatropha* seed development. Overexpressing *SWEET15* in upland cotton reduces fibre length, seed size and oil content (Le et al. 2025). In this study, the expression of *SWEET10* was down regulated in *rSPL9* transgenic plants.

Biodiesel quality is mainly determined by the FA composition of vegetable oils (Feng et al. 2020; Ramos et al. 2009; Tang et al. 2022). Since polyunsaturated FAs reduce OS of biodiesel, vegetable oils rich in monounsaturated FAs with low DU are preferable to those containing polyunsaturated FAs (Feng et al. 2020; Ye et al. 2013). A gene silencing approach has been applied to improve the oil quality of *Jatropha* seeds (Qu et al. 2012; Ye et al. 2013). Silencing of *JcFAD2‐1* encoding a fatty acid desaturase 2, which catalyses the conversion of oleic acid (C18:1) to linoleic acid (C18:2) (Tao et al. 2019), showed a significant increase in oleic acid content by more than 78% (Qu et al. 2012). In this study, the *rJcSPL9* transgenic *Jatropha* exhibited a significant increase in oleic acid (C18:1) and a corresponding decrease in linoleic acid (C18:2) in the seed oil (Figure 6F and Figure S9E), which resulted in a significantly decreased DU of the seed oil. Therefore, an improved biodiesel with higher OS was detected in seed oil of *rJcSPL9* transgenic *Jatropha* than in that of WT (Figure 6G and Figure S9F).

In conclusion, we show that *JcSPL9* is involved in the regulation of both seed yield and oil productivity in *Jatropha*, and for the first time, we demonstrate, by using the *rJcSPL9* and *JcmiR156a* transgenic *Jatropha*, that *SPL9* has a novel function in regulating fatty acid biosynthesis and lipid accumulation in seeds. The results suggest a possible method for developing high‐yield, high‐oil content transgenic germplasms to enhance seed yield and oil traits in woody oilseed plants. The breeding biotechnology, which employs a mutant of miR156 target sequence, can also be applied to developing new varieties of other oil crops. Due to the substantial regulatory hurdles in transgenic variety registration mandating long‐term safety assessments, regulatory approval for these new varieties has yet to be secured. Despite this, the technology and methodology could point to a promising new direction for agriculture. The cross‐disciplinary integration of molecular precision breeding biotechnologies with artificial intelligence and robotics will reshape future market landscapes, ignite industrial vitality, and foster breakthrough products. *JcSPL9* represents a promising tool for increasing seed yield, oil productivity, and quality in *Jatropha*, with potential applications to other oilseed crops. Further studies should elucidate the molecular mechanisms underlying *JcSPL9*‐mediated regulation of fatty acid and lipid metabolism in seeds. A new function of *SPL9* was also found in *Arabidopsis*, i.e. cuticular wax regulation by the miR156‐*SPL9* module in *Arabidopsis* (Clark et al. 1993; Huang et al. 2024) and enhanced soluble sugar accumulation in cassava roots through *rMeSPL9* overexpression (Li, Cheng, et al. 2022).

## Author Contributions

Mingyong Tang designed and performed the experiments, analysed the data, and wrote the paper. Xue Bai performed the experiments, analysed the data, and revised the paper. Yaoping Xia and Ping Huang helped collect the data. Zeng‐Fu Xu conceived the experiments and revised the manuscript. All authors reviewed and approved the final manuscript.

## Funding

This work was supported by the Natural Science Foundation of China (32371836), the Guangxi Specific Project for Science and Technology Bases and Talents (AD23026337), and the Natural Science Foundation of Yunnan Province (202501AS070081, 202205AC160030, 202401AT070225).

## Conflicts of Interest

The authors declare no conflicts of interest.

## Supporting information


**Data S1:** pbi70558‐sup‐0001‐Supinfo.docx.

## Data Availability

No new sequence data was published in the present paper. sequence data included in our manuscript can be obtained from the publicly available genome of *Jatropha curcas* (https://www.ncbi.nlm.nih.gov/bioproject/PRJNA694573/). Under the following accession numbers: *JcSPL9* (XM_012232354); *JcmiR156a* (XR_002284867); *JcmiR172* (XR_002283652); *JcAP1*(KR013222); *JcLFY* (XM_012235184); *JcSOC1* (XM_012228124); *JcFUL* (XP_020537425), *JcACTIN1* (NM_112764); *JcDGAT1* (NM_001305997); *JcDGAT2* (NM_001306044); *JcOLEOSIN* (JQ806305); *JcWRI1* (NM_001306018).
