# Supplementary material for: SBP‐Box Transcription Factor JcSPL9 Regulates Both Seed Yield and Oil Content in the Biofuel Plant Jatropha curcas
Source: Plant Biotechnol J. 2026 Jan 23;24(5):3125–40. doi: 10.1111/pbi.70558 (PMC13110145; doi:10.1111/pbi.70558)
Supplement: Supplementary file 1 — Data S1: pbi70558‐sup‐0001‐Supinfo.docx. [file PBI-24-3125-s001.docx]

*Jatropha curcas* SBP-box transcription factor JcSPL9 is involved in the regulation of both seed yield and oil contents

Mingyong Tang^1,^*, Xue Bai^1,2^, Yaoping Xia^1,^, Ping Huang^1,2^, and Zeng-Fu Xu^1, 3^^,^*

^1^CAS Key Laboratory of Tropical Plant Resources and Sustainable Use, Xishuangbanna Tropical Botanical Garden, Chinese Academy of Sciences, Menglun, Yunnan 666303, China

^2^University of Chinese Academy of Sciences, Beijing 100049, China

3State Key Laboratory for Conservation and Utilization of

Subtropical Agro-Bioresources, College of Forestry, Guangxi University, Guangxi, Nanning 530004, China

^*^Author to whom correspondence should be addressed; E-mail: [tangmingyong@xtbg.ac.cn](mailto:tangmingyong@xtbg.ac.cn), [zfxu@gxu.edu.cn](mailto:zfxu@gxu.edu.cn)


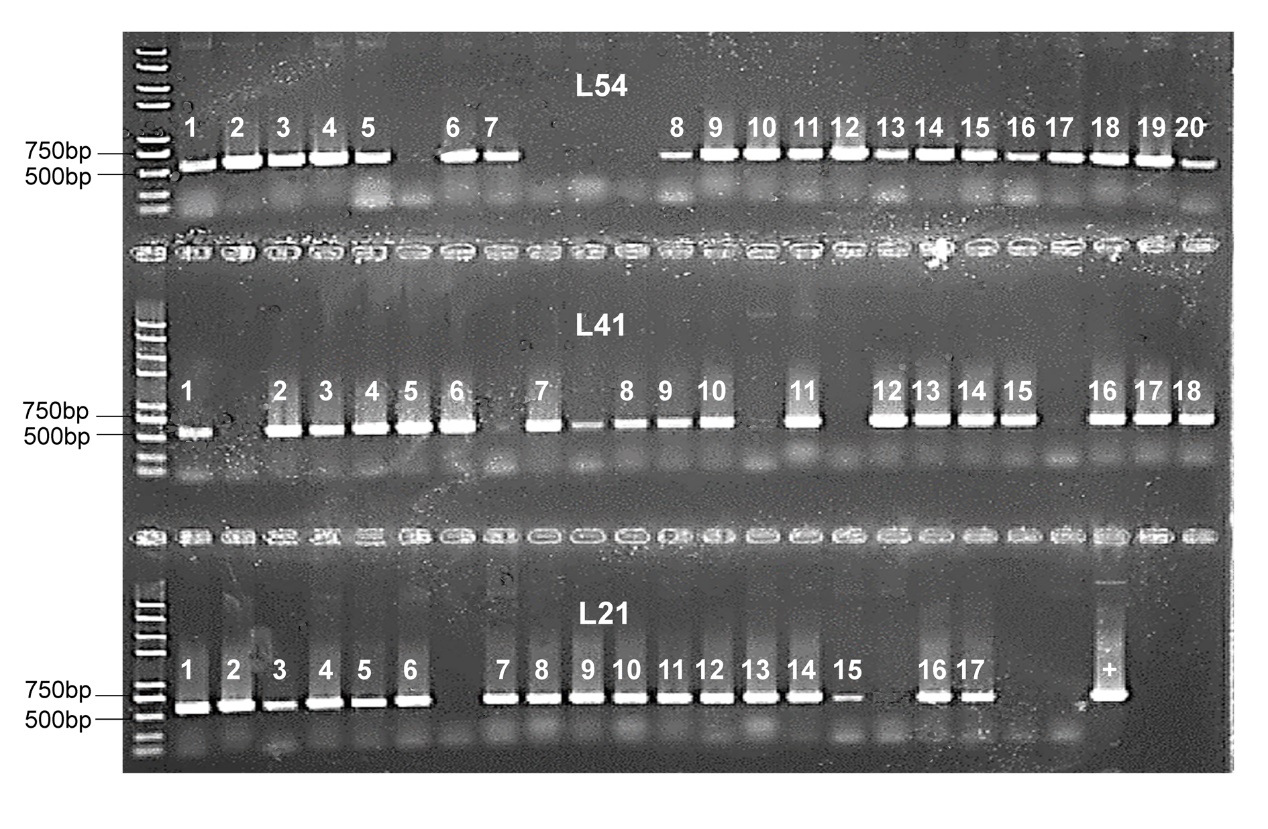


**Figure S1. Identifying the T1 positive *Jatropha* seedlings by PCR**. The seeds got from T0 plants were used to germinate T1 seedings. The caMV35S promoter sense primer XT126 and *JcSPL9* qRT-PCR antisense primer XA643 were used. The 625 bp fragment product was amplified, the binary vector was used as positive control (+).


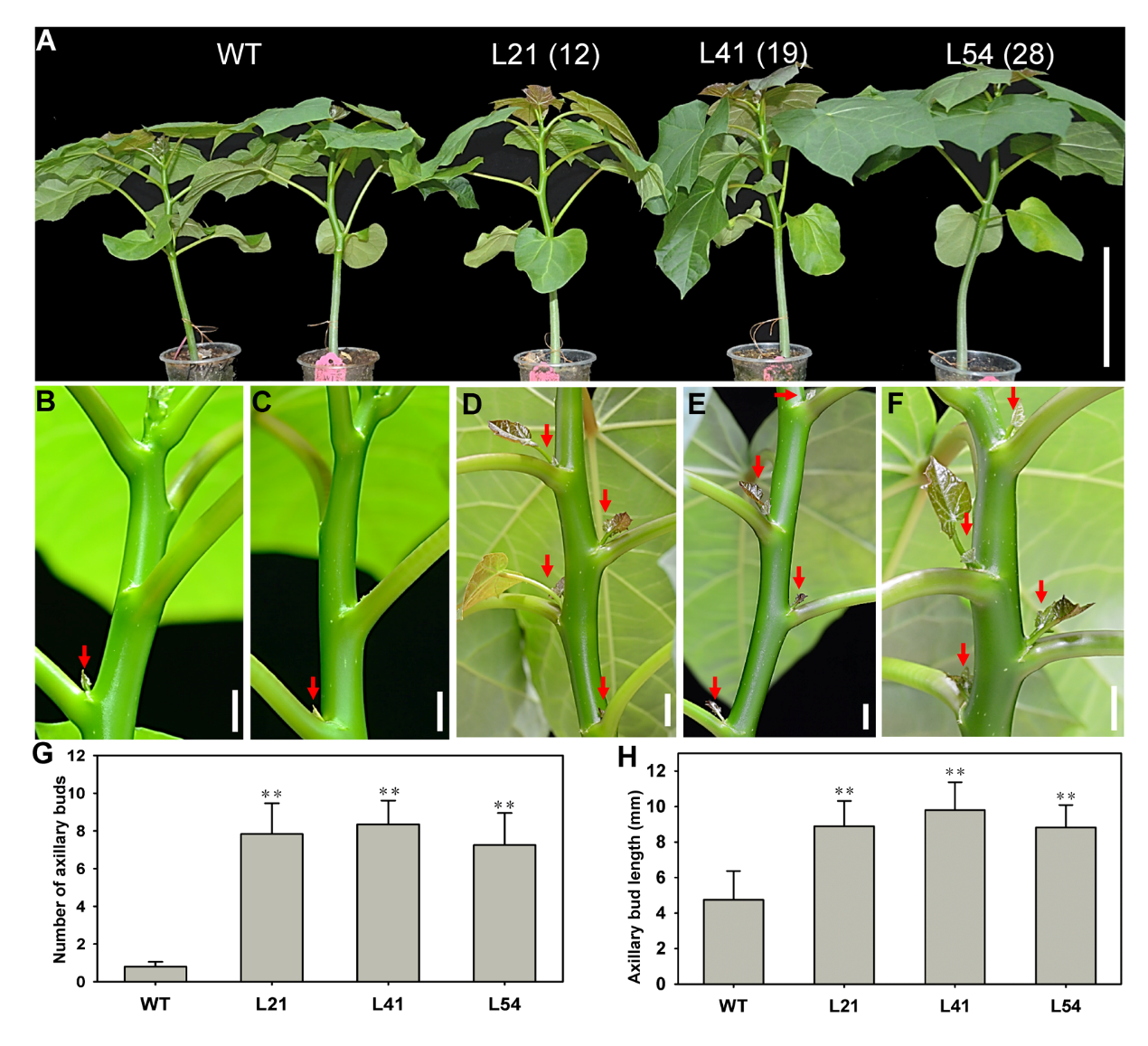


**Figure S2 *JcSPL9* promoted axillary bud outgrowth in *Jatropha.*** (A) Phenotypes of two-month-old WT and three independent T1 transgenic seedlings. (B-F) The shoots of WT (B, C) and T1 transgenic L21 (D), L41 (E), and L54 (F); the arrows indicate the axillary buds; (G, H) Analysis of axillary bud numbers (G) and axillary bud lengths (H). ** indicates P < 0.01. Bars = 10 cm (A) and 1 cm (B-F).


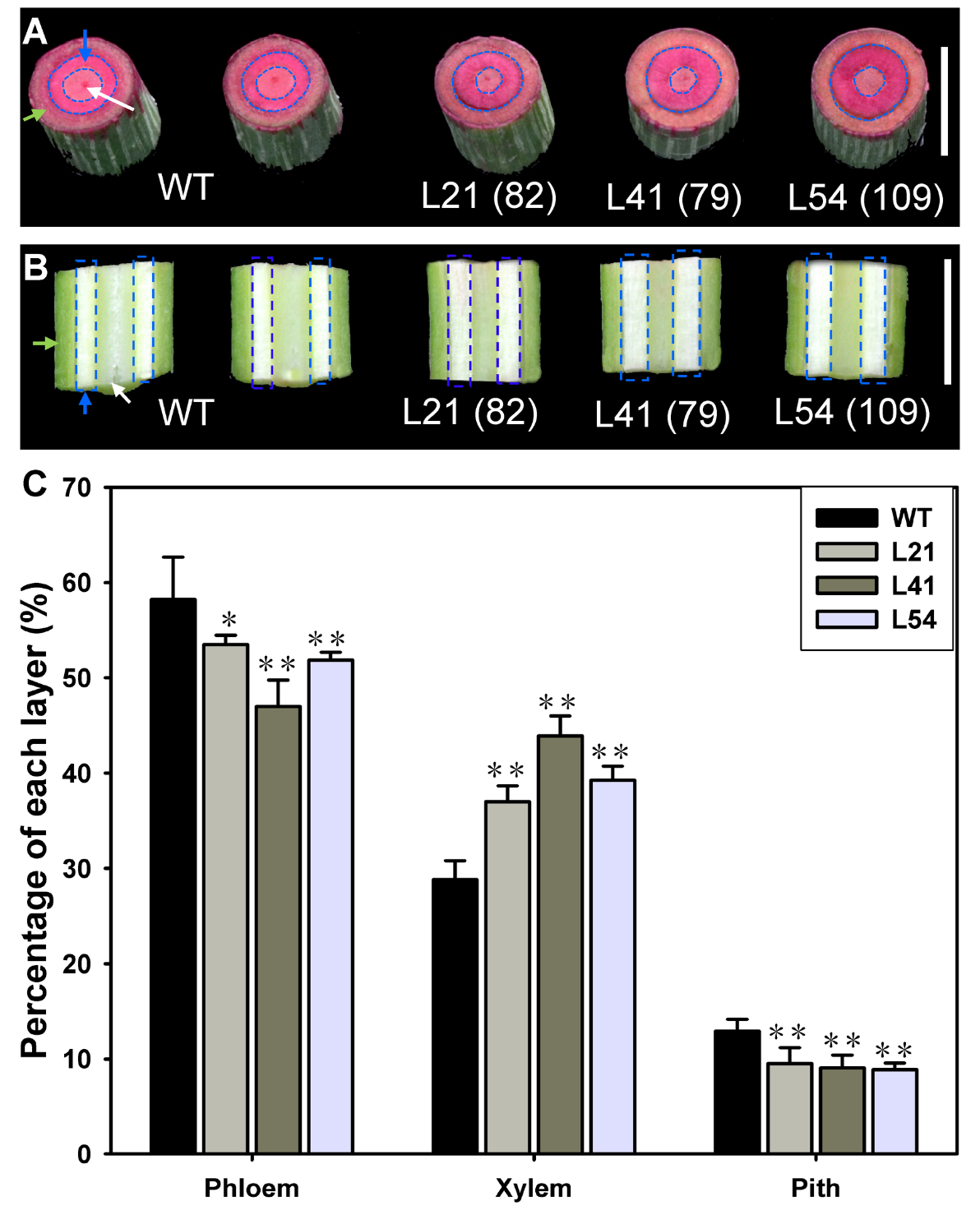


**Figure S3 The *rJcSPL9* transgenic *Jatropha* exhibited thickened xylems.** (A) Cross-section of the stems of 2-month-old seedlings of WT plants and the transgenic T1 plants L21, L41 and L54. The stems were stained with 5% potassium permanganate solution and 5% red ink. (B) Longitudinal sections of stems of 2-month-old seedlings of WT plants and the transgenic T1 plants L21, L41 and L54. (C) Analysis of the percentages of phloem, xylem, and pith areas. The values are the means ± standard deviations. * Statistically different from the control at the 5% level, ** Statistically different from the control at the 1% level. The error bars indicate the standard deviations for 25 plants. Bars = 1 cm. the green arrows indicate phloem, the blue arrows indicated xylem, the white arrow indicate pith.


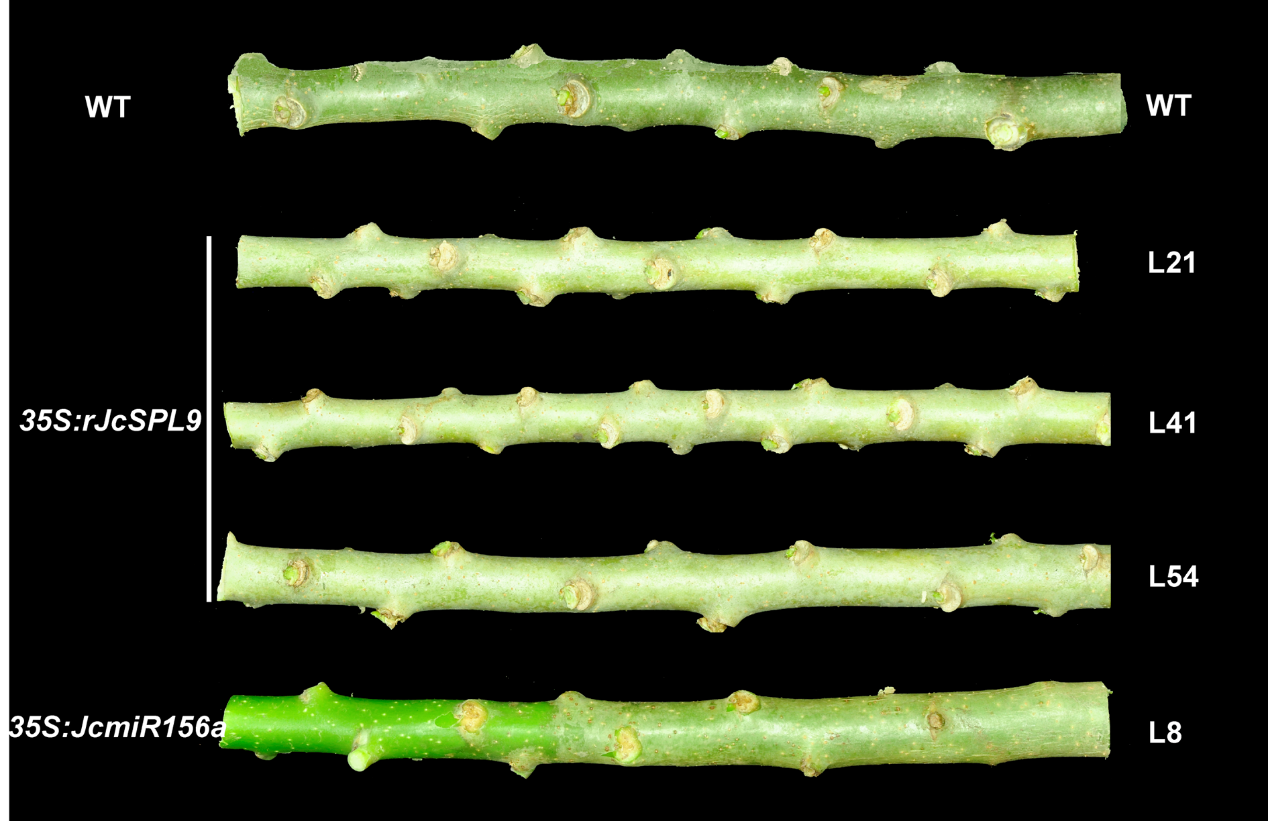


**Figure S4 The *35S:rJcSPL9* transgenic *Jatropha* exhibited obvious epidermal wax.** The stems 50 cm distance from shoot apices was collected from 2-year-old WT, *35S:rJcSPL9,* and *35S:JcmiR156a* plants.


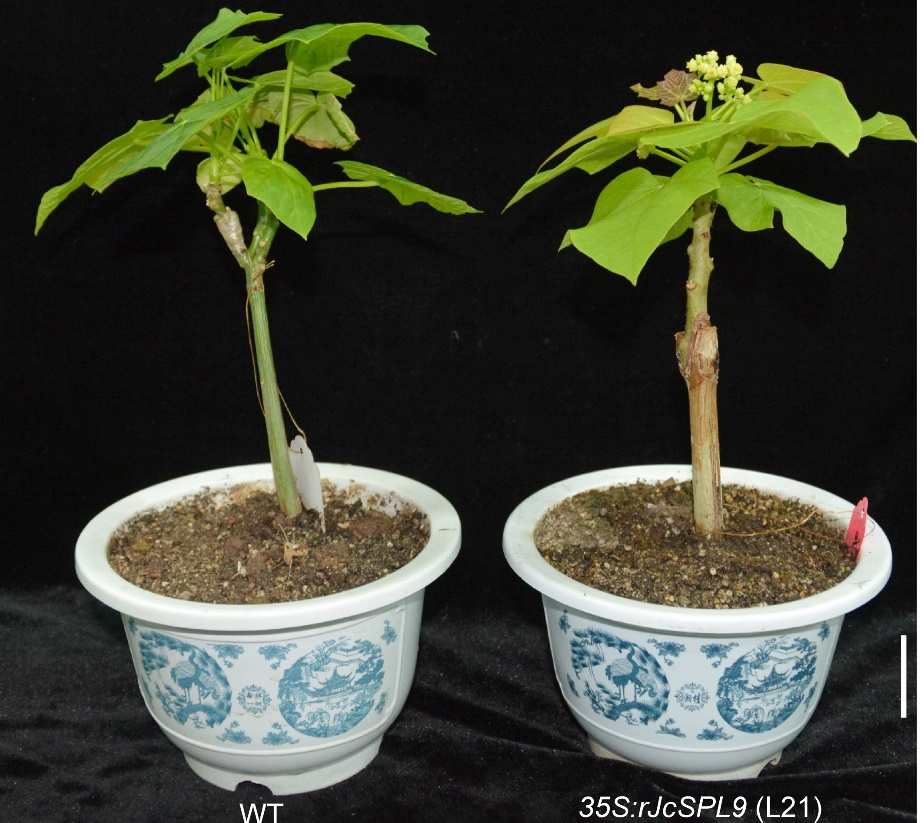


**Figure S5 *JcSPL9* promoted flowering in *Jatropha*.** Three-month-old WT and *35S:rJcSPL9* (L21) transgenic plants grown in the climate chamber, flowers produced in the transgenic plants. WT and transgenic shoots were grafted on to one-month-old WT rootstocks*.* bar = 10 cm


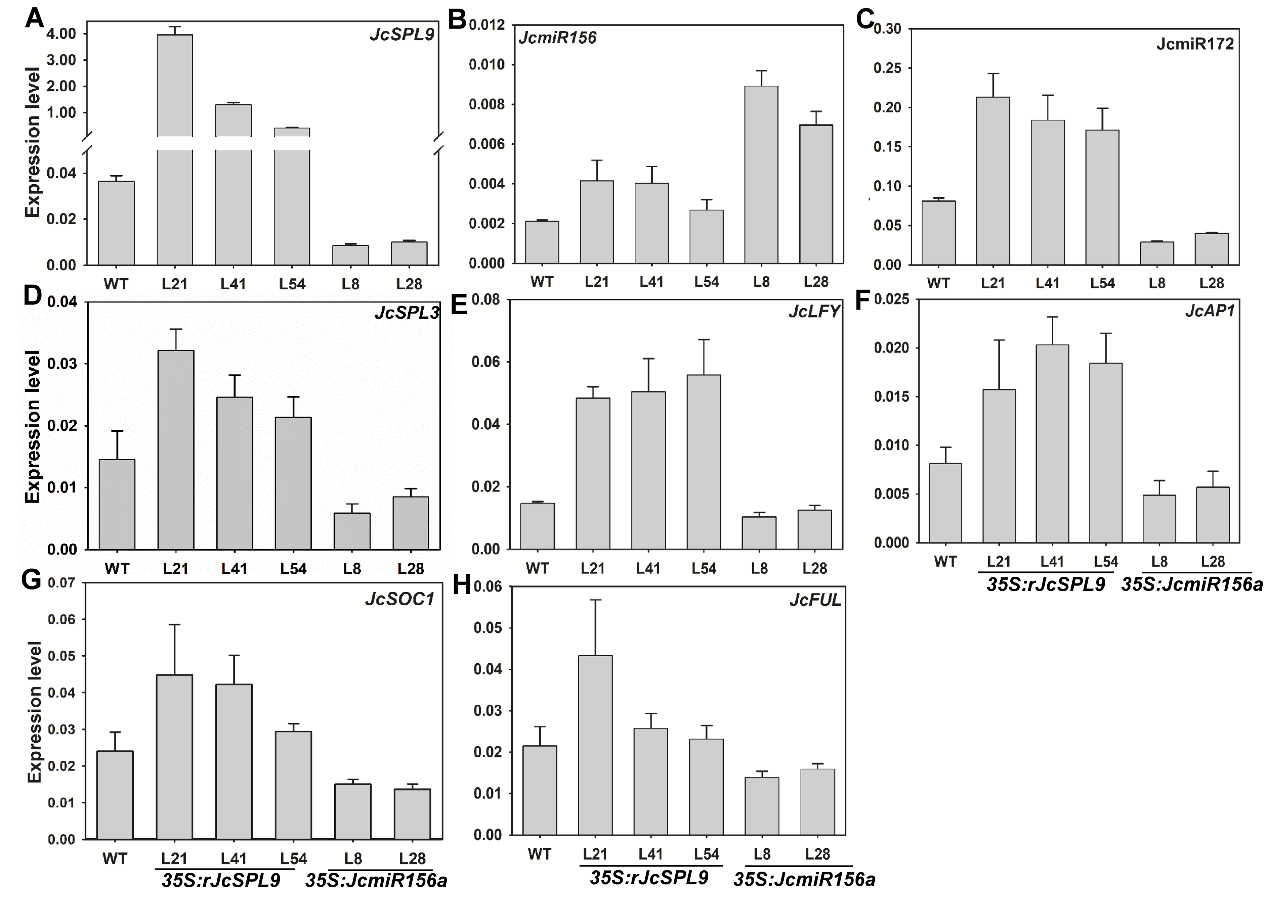


**Figure S6 Quantitative RT-PCR analysis of expression of *JcSPL9* and other flowering-related genes in WT and transgenic *Jatropha*.** (A-B) Expression levels of *JcSPL9* and JcmiR156 in flower buds of 8-month-old WT, *35S:rJcSPL9* transgenic *Jatropha* L21, L41, L54, and *35S:JcmiR156a* transgenic plant were detected. (C-H) Expression abundances of JcmiR172, *JcSPL3, JcLFY, JcAP1, JcSOC1,* and *JcFUL* were detected respectively. RNA samples were extracted from flower buds of 8-month-old WT, *35S:rJcSPL9* and *35S:JcmiR156a* transgenic T1 plants. *JcActin1* gene as a reference. The y-axes represent the relative expression levels of mRNAs against reference gene. Error bars indicate standard deviations for three biological replicates.


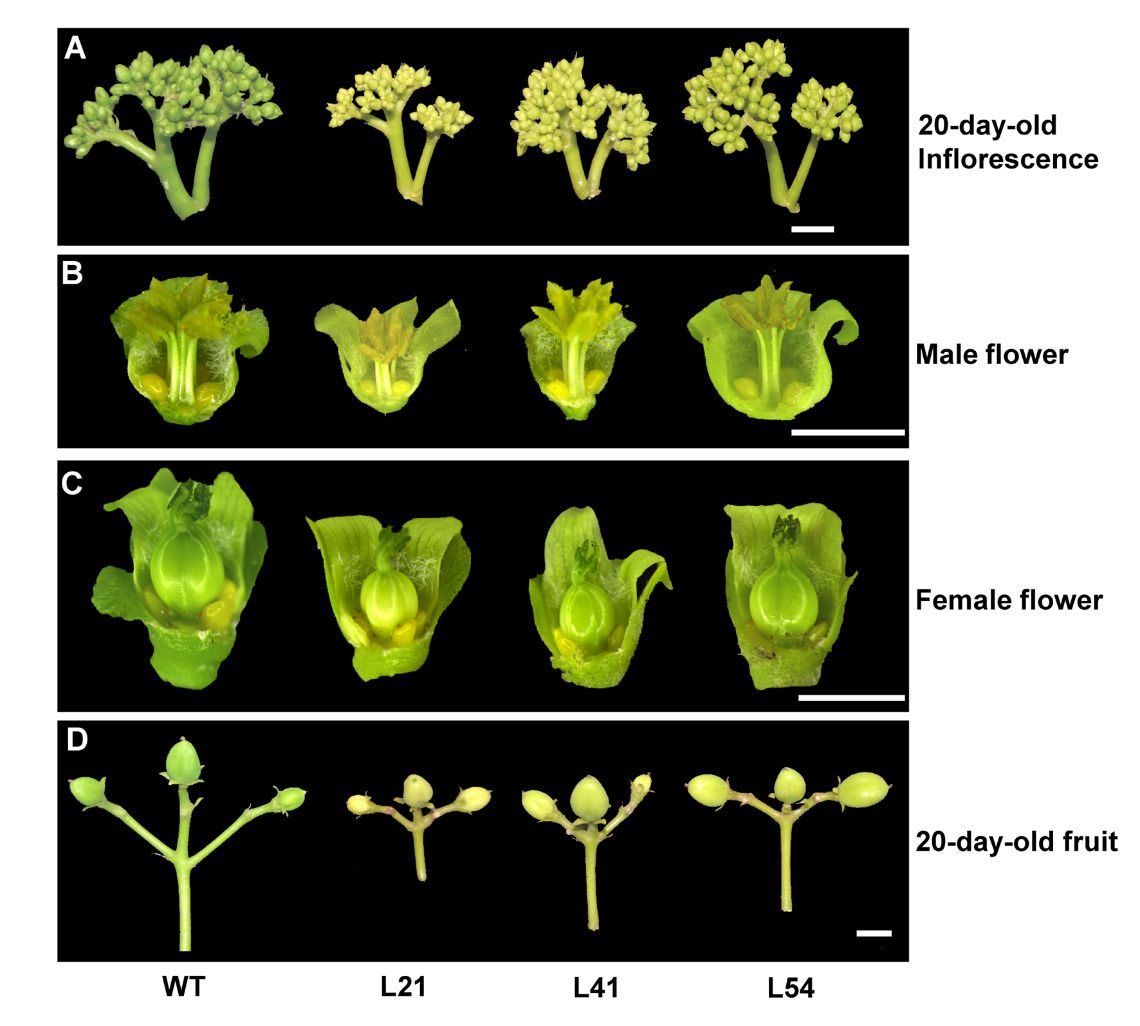


**Figure S7 The *35S:rJcSPL9* transgenic *Jatropha* exhibited smaller reproductive** **organs.** (A) 20-day-old Inflorescence from WT and T1 transgenic L21, L41, and L54, the inflorescence and flower buds grown in the transgenic inflorescence were smaller than WT; (B) Bloomed male flowers from WT and T1 transgenic plants L21, L41, and L54; (C) Bloomed female flowers from WT and T1 transgenic plants L21, L41, and L54; (D) 20-day-old fruits collected from WT and T1 transgenic plants L21, L41, and L54; bar = 1 cm.


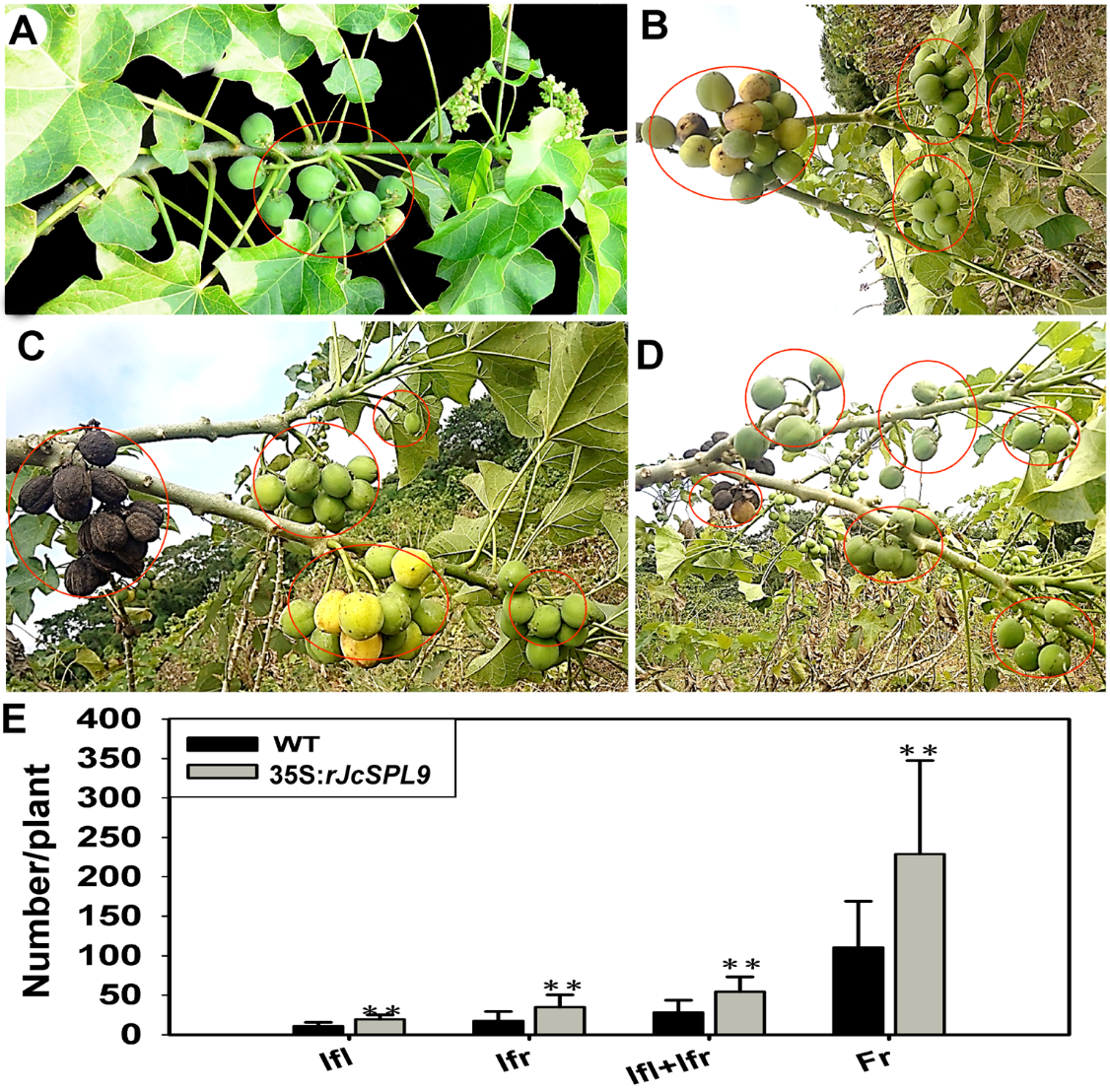


**Figure** **S8 Both the number of** **inflorescences and infructescences increased in T0 *rJcSPL9* transgenic *Jatropha.*** (A) Only one infructescence and one inflorescence developed on a branch of WT. (B) Four infructescences are shown on a branch of *rJcSPL9* transgenic *Jatropha* L21. (C) Five infructescences are shown on a branch of *rJcSPL9* transgenic *Jatropha* L41. (D) Six infructescences are shown on a branch of *rJcSPL9* transgenic *Jatropha* L54. (E) Statistical analysis of inflorescence (Ifl) and infructescence (Ifr) numbers and fruit (Fr) numbers per plant. The values are the means ± standard deviations. The asterisks denote significant differences in comparison to WT plants (Student’s t-test: **, P < 0.01). The transgenic plants were planted in the filed in the summer of 2014. The infructescences are indicated with red circles, and the inflorescence is indicated with a dashed red circle.


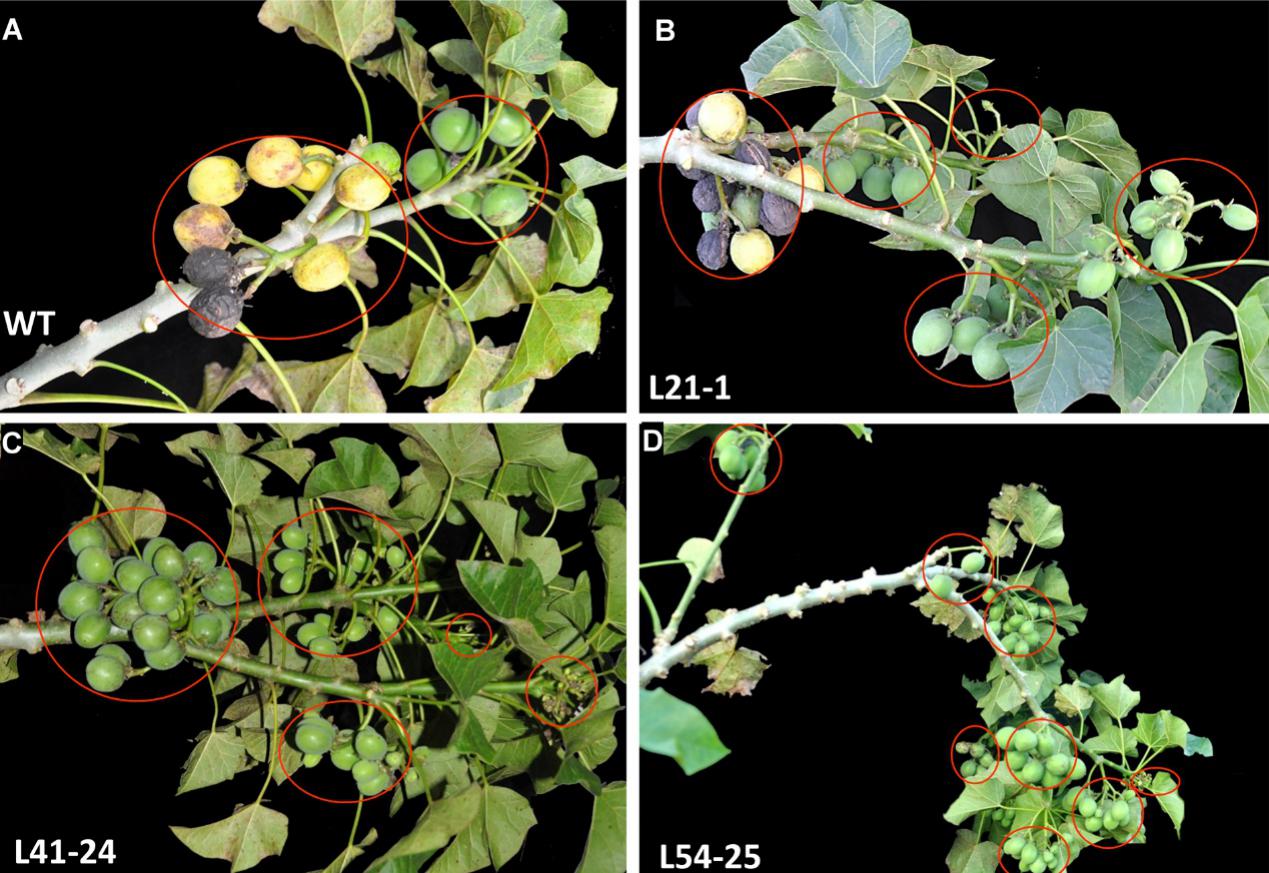


**Figure** **S9 The infructescence number increased in T1 *rJcSPL9* transgenic *Jatropha.*** Comparison of the infructescences on the primary branches between WT and *rJcSPL9* transgenic *Jatropha* (L21-1, L41-24, and L54-25). The infructescences are indicated with red circles.


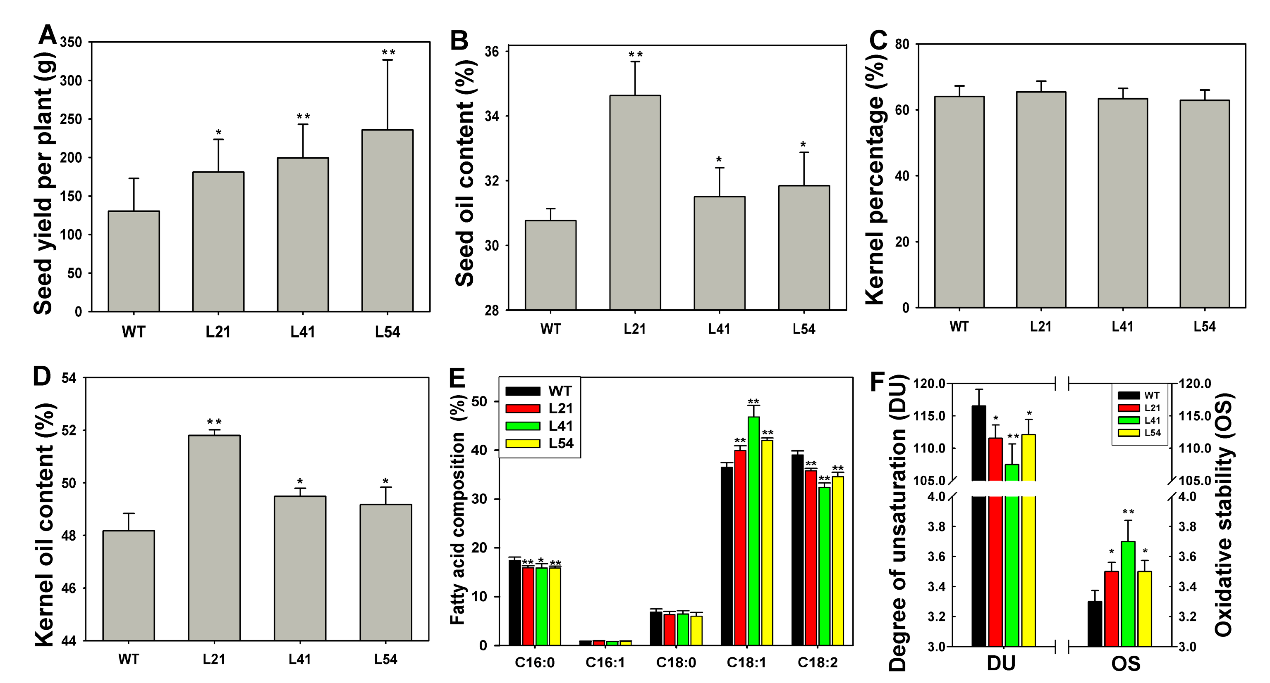


**Figure S10 Seed yield and oil content and fatty acid composition in T1 *rJcSPL9* transgenic plants.** (A, B) Comparison of seed yield (A) and oil content (B) between wild-type (WT) and the *rJcSPL9* transgenic *Jatropha* (L21, L41, and L54). (C) Comparison of the kernel percentage of dry seeds between WT and the *rJcSPL9* transgenic *Jatropha* (L21, L41, and L54). (D) Comparison of kernel oil content between WT and *rJcSPL9* transgenic *Jatropha* (L21, L41, and L54). (E）Comparison of the fatty acid composition of seed oil from WT and the *rJcSPL9* transgenic *Jatropha.* (F) Comparison of the degree of unsaturation (DU) and oxidative stability (OS) of seed oil from WT and the *rJcSPL9* transgenic *Jatropha.* Values are means ± standard deviations, which were calculated from at least 100 seeds independently collected from WT and transgenic plants. Asterisks denote significance compared with WT plants (Student’s t-test: *, P < 0.05; **, P < 0.01).


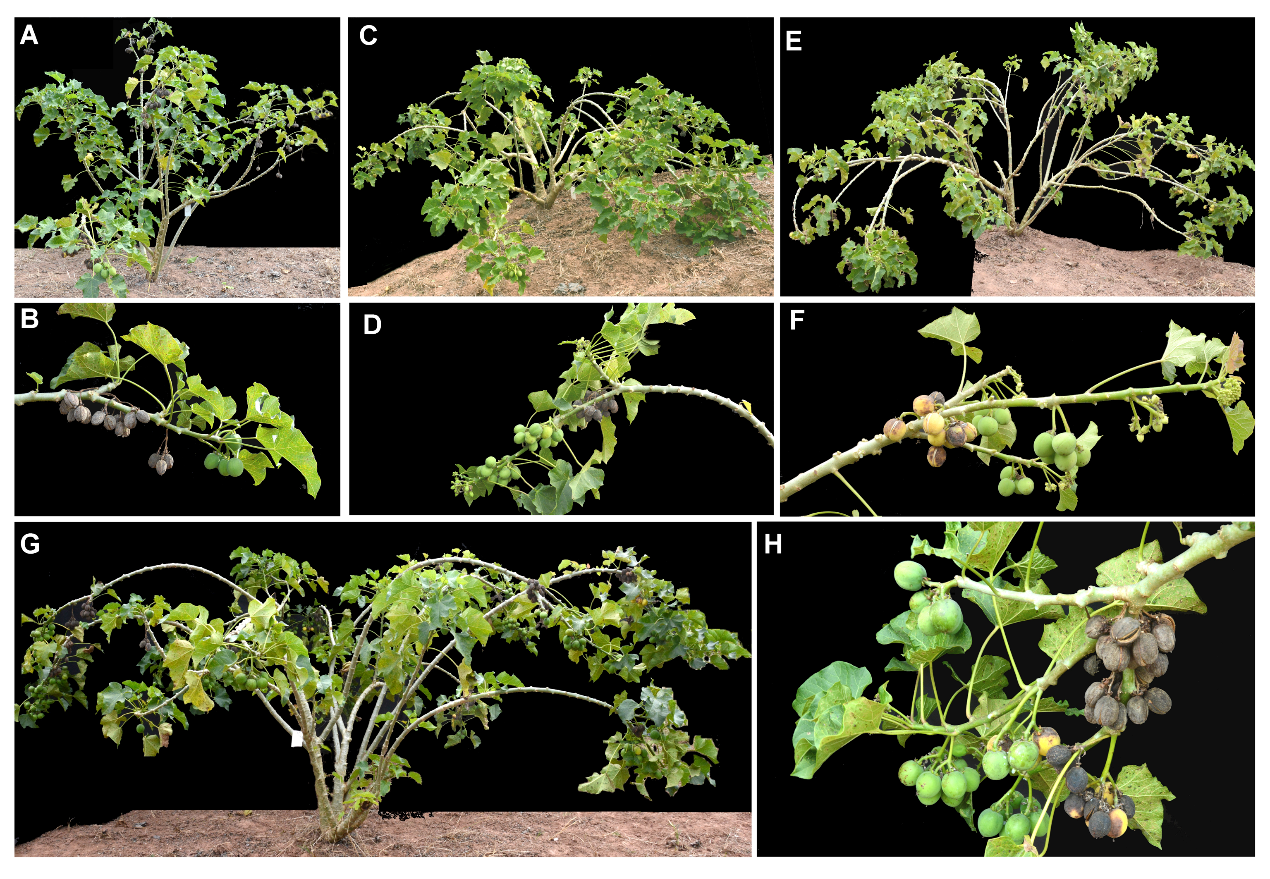


**Figure** **S11 The infructescence number increased in T1 *rJcSPL9* transgenic *Jatropha* in the third year***.* (A) Three-year-old WT *Jatropha* plant; (B) A branch of WT plant. Only three infructescence produced in a branch; (C) Three-year-old T1 *rJcSPL9* transgenic plant (L21); (D) A branch of T1 *rJcSPL9* transgenic plant (L21). Five infructescence produced in a branch; (E) Three-year-old T1 *rJcSPL9* transgenic plant (L41); (F) A branch of T1 *rJcSPL9* transgenic plant (L41). Six infructescence produced in a branch; (G) Three-year-old T1 *rJcSPL9* transgenic plant (L54); (H) A branch of T1 *rJcSPL9* transgenic plant (L51). Six infructescence produced in a branch.


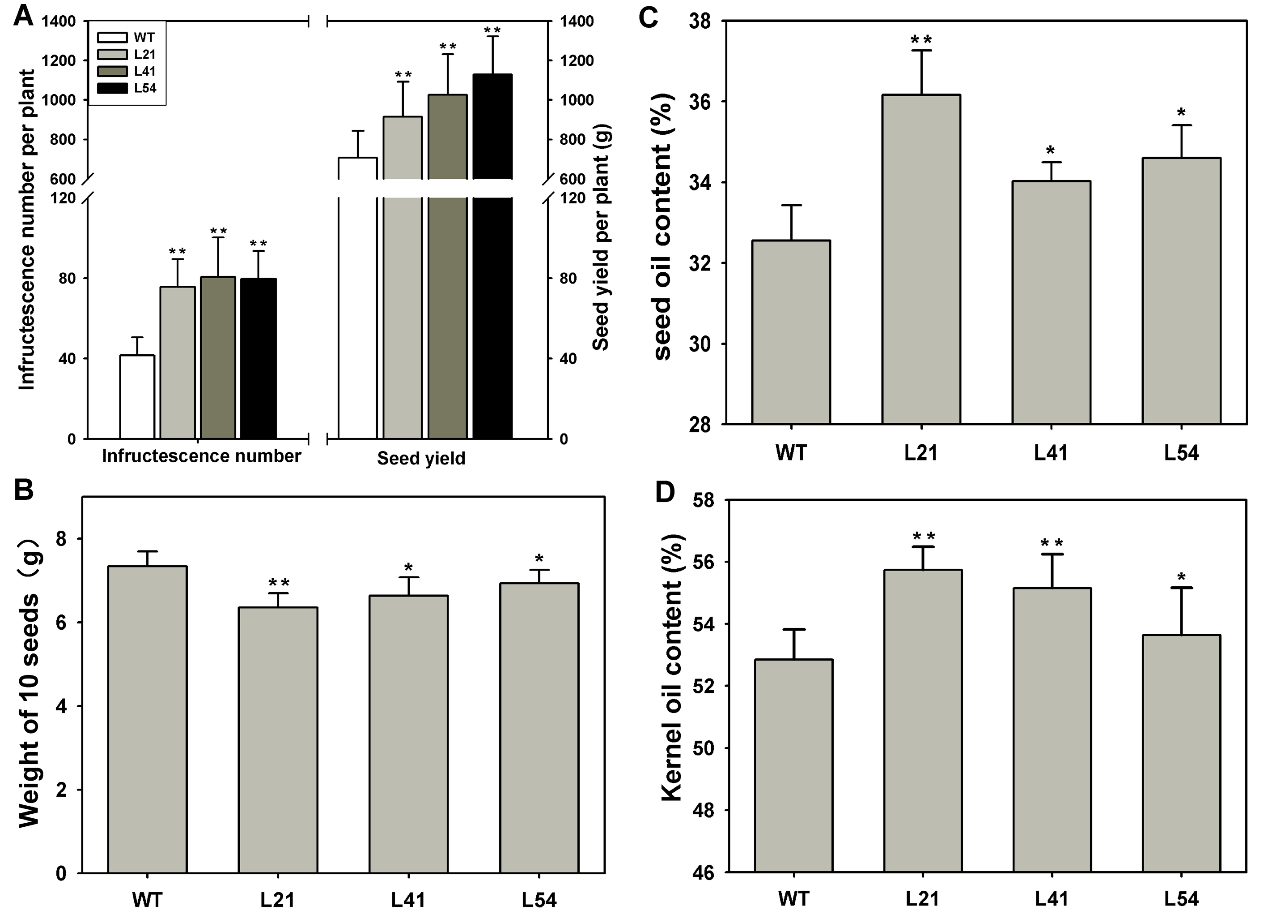


**Figure** **S12 Seed yield and oil content in *rJcSPL9* transgenic plants were significantly increased in the third year.** Comparison of infructescence number and seed yield of each plant (A), weight of 10 seeds (B), seed oil content (C), and kernel oil content (D) between wild-type (WT) and the *rJcSPL9* T1 transgenic *Jatropha* (L21, L41, and L54) in the third year, ten plants of each genotype were used to analysis seed yield and infructescence number (Student’s t-test: *, P < 0.05; **, P < 0.01).


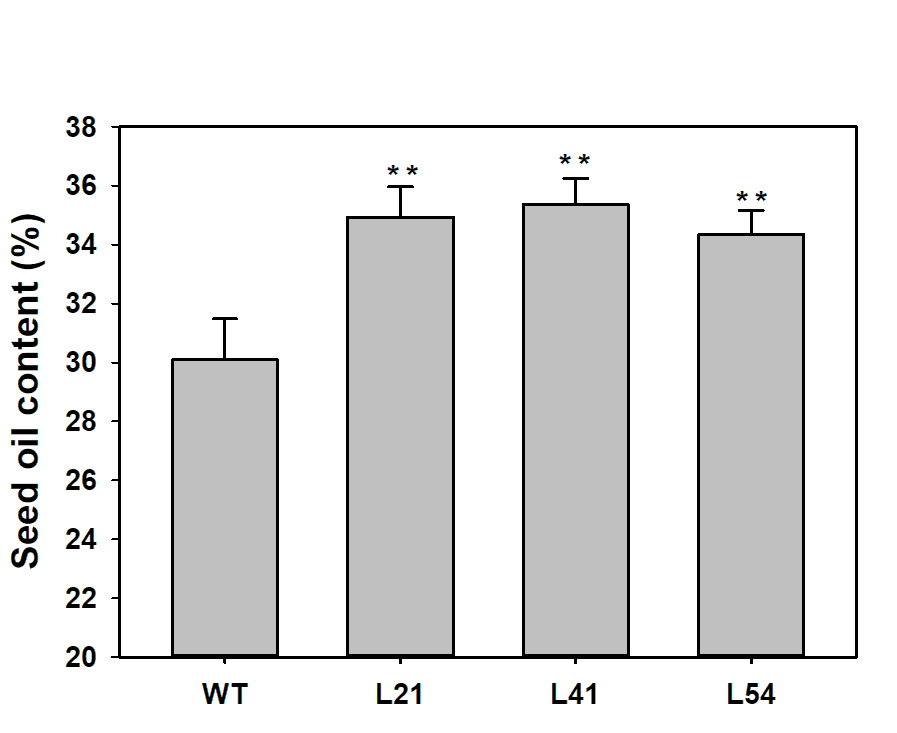


**Figure S13 Oil content in *rJcSPL9* transgenic plants were significantly increased in the fourth year.** Comparison of seed oil content between wild-type (WT) and the *rJcSPL9* T1 transgenic *Jatropha* (L21, L41, and L54) in 2024, the plants used in this study were planted in the November of 2020, ten plants of each genotype were used to analysis seed oil content (Student’s t-test: **, P < 0.01).


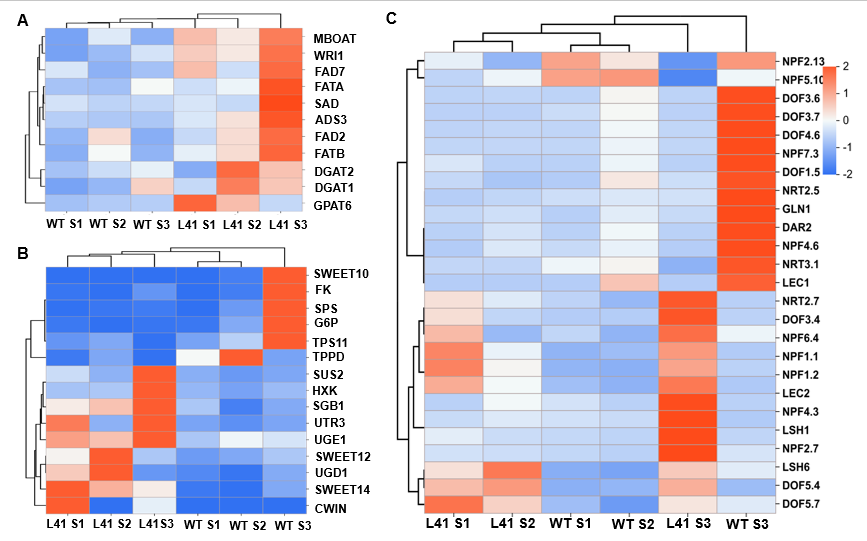


**Figure S14 Heat map showing expression profiles of genes involved in oil metabolism (A), sugar metabolism (B), and protein metabolism (C) pathways in wild-type (WT) and *rJcSPL9* transgenic kernel**. Transcriptional profiles are derived from RNA-seq data for differentially expressed genes (DEGs) during the late stages of seed development (S1: 5 weeks, S2: 6 weeks, S3: 7 weeks after pollination) in WT and the *rJcSPL9* transgenic line L41. Data were shown as log2(normalized FPKM).


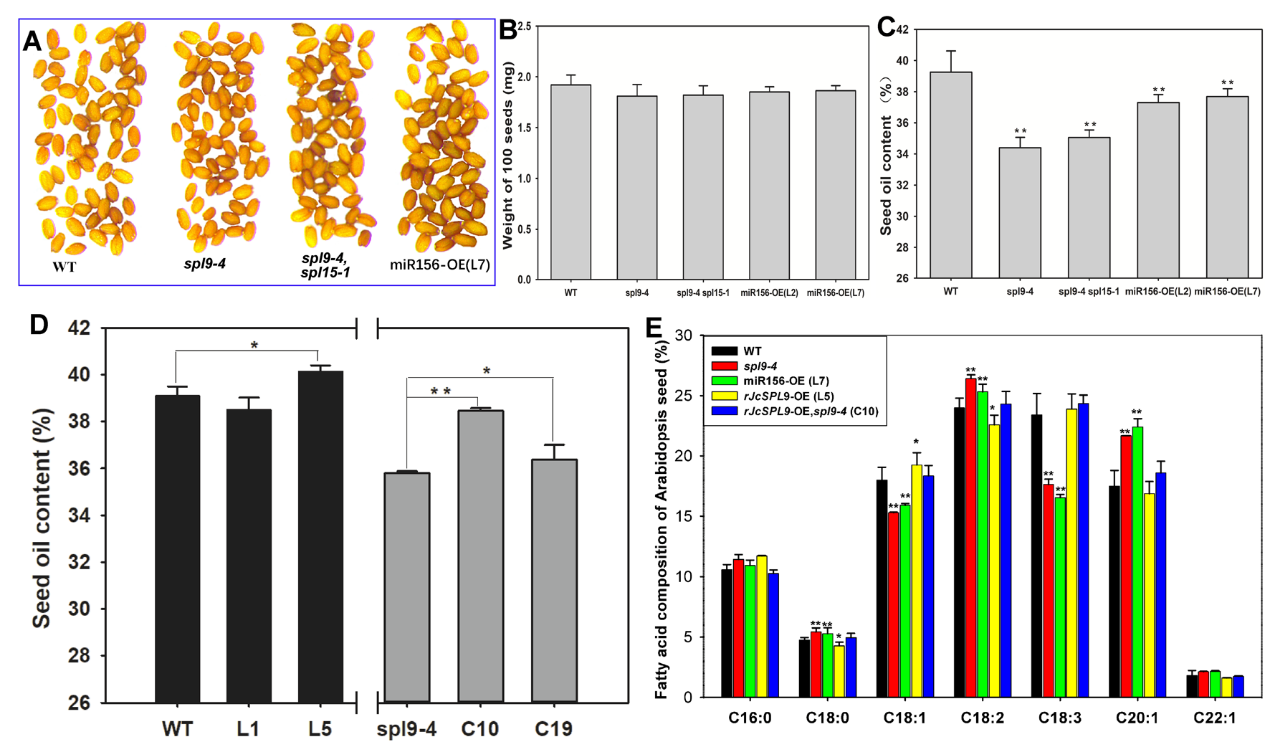
**Figure S15 Seed size, oil content and fatty acid composition in WT, *spl9* mutants, and *JcmiR156a* overexpressing (miR156-OE) *Arabidopsis* seeds.** (A) Seed morphology; (B) Seed weight. The weight of 100 seeds were determined with six samples of 100 seeds; (C) Seed oil content. Seed oil was extracted by Soxhlet extraction using about 10 g mature seeds; (D) Fatty acid composition of seed oil; (E-F) Seed size. Seed length (E) and width (F) were determined with 30 seeds. Error bars indicate standard deviations for three biological replicates. Asterisks denote significance compared with WT plants (Student’s t-test: *, P < 0.05; **, P < 0.01).

**Table S1 Flowering time and rosette leave number of WT and different kinds of *JcSPL9* transgenic *Arabidopsis***

| Genotype | N | Rosette leave | Flowering time （day） |
| --- | --- | --- | --- |
| WT | 20 | 12.4 ± 1.3^b^ | 26.9 ± 1.6 ^b^ |
| *35S:rJcSPL9* (L18) | 15 | 8.5 ± 0.8^d^ | 16.5 ± 1.4^d^ |
| *35S:JcmiR156a* (L7) *× 35S:rJcSPL9*(L18) | 13 | 9.1 ± 0.9^d^ | 17.4 ± 1.6^d^ |
| *35S:JcSPL9* (L11) | 15 | 10.2 ± 1.0^c^ | 19.8 ± 1.5^c^ |
| *35S:JcmiR156a* (L7) *× 35S:JcSPL9* (L11) | 14 | 13.1 ± 1.5^b^ | 27.3 ± 1.7^b^ |
| *35S:JcmiR156a* (L7) | 15 | 19.5 ± 1.9^a^ | 42.5 ± 2.7^a^ |

WT and five kinds of transgenic *Arabidopsis* grown under LD growing conditions (16h light/8h dark) were used to analysis the rosette leaves and flowering time. N, plants numbers; the rosette leaves and flowering times were indicated as mean ± standard deviation. Different letters indicated difference at 5% level. In this study 35 lines of *35S:rJcSPL9* transgenic *Arabidopsis* were obtained, in which 20 lines exhibited an early flowering phenotype; the early flowering percentage is 57%, the earliest line L18 was chosen to analyze flowering time and hybrid experiment. And 32 lines of *35S:JcSPL9* transgenic *Arabidopsis* were obtained, in which 5 lines exhibited an early flowering phenotype; the early flowering percentage is 16%; the earliest line L11 was chosen to analyze flowering time and hybrid experiment.

**Table S2 Overexpression of *rJcSPL9* increased flower numbers in transgenic *Jatropha*.**

| **Line** | **No. of plants** | **Flowering time (day)** | **Female flowers per inflorescence** | **Male flowers per inflorescence** | **Female : Male** |
| --- | --- | --- | --- | --- | --- |
| WT | 15 | 193.41 ± 26.34 | 8.75 ± 1.42 | 143.63 ± 23.45 | 1:16.75 ± 3.72 |
| L21 | 15 | 168.53 ± 30.28* | 13.73 ± 1.83** | 200.27 ± 14.64** | 1:14.68 ± 2.23 |
| L41 | 21 | 173.46 ± 32.47 | 14.12 ± 3.56** | 220.75 ± 31.15** | 1:16.81 ± 4.63 |
| L54 | 19 | 175.64 ± 28.14 | 12.83 ± 3.56** | 181.28 ± 31.15** | 1:15.61 ± 3.27 |

The Flowering time and flower number of WT plants and T1 plants of three independent *rJcSPL9* overexpression lines (L21, L41 and L54) grown in the field were analysed. The number are presented as the means ± standard deviations. * Significantly different from the control at the 5% level. ** Significantly different from the control at the 1% level.

**Table S3 Primers used in this study. F, forward primer; R, reverse primer.**

| **Gene template** | **Accession number** | **Purpose** | **Primer name** | **Primer sequence** |
| --- | --- | --- | --- | --- |
| *JcSPL9* | XM_012232354 | Full length | XA39 F | GGGGTACCCCGTCAGTATAGAGTCTTCGCCAG |
|  |  |  | XA40 R | GCGTCGACGTTGCCCCAACATAAAAATACACG |
| *JcSPL9* | XM_012232354 | Mutation | XA41 F | AGAGAGCAGTGACAGTGCGCAGCTTGAGTCAGCAATGC |
|  |  |  | XA42 R | TGCGCACTGTCACTGCTCTCTAATCAACCATGGGGCTC |
| *JcmiR156a* | XR_002284867 | Full length | XA43 F | GGGGTACCCCGCACCATTATTTCCCATTACCATC |
|  |  |  | XA44 R | GCGTCGACGTCGTCTGATCTTCTGGGACACAGAAATT |
| *JcSPL3* | XM_012236245 | qRT-PCR | XA640 F | TTGGTGGATTAGGGTTTGGA |
|  |  |  | XA641 R | CACTTTATGGCGTCTGTGGTAT |
| *JcSPL9* | XM_012232354 | qRT-PCR | XA642 F | TTTGACCAAGGAAAACGAAG |
|  |  |  | XA643 R | CATAAAGTGTTGAAGAGAGACGG |
| *JcmiR156* | XR_002284867 | Reverse transcription | XA819 R | GTCGTATCCAGTGCAGGGTCCGAGGTATTCGCACTGGATACGACGTGCTC |
| *JcmiR156* | XR_002284867 | qRT-PCR | XA820 R | GCGGCGGTGACAGAAGAGAGT |
|  |  |  | XA823 F | GGCGGCGAGAATCTTGATGATG |
| *JcmiR172* | XR_002283652 | Reverse transcription | XA822 R | GTCGTATCCAGTGCAGGGTCCGAGGTATTCGCACTGGATACGACATGCAG |
| *JcmiR172* | XR_002283652 | qRT-PCR | XA823 F | GGCGGCGAGAATCTTGATGATG |
|  |  |  | XA821 R | GTGCAGGGTCCGAGGT |
| *JcAP1* | KR013222 | qRT-PCR | XA311 F | TAACAGACTCAAGGCGAAGGT |
|  |  |  | XA312 R | AGTTGGTTGTTTCTTGCTCGG |
| *JcLFY* | XM_012235184 | qRT-PCR | XT655 F | GGATAAGATACTACACAGCAGCGA |
|  |  |  | XT656 R | TAACCCTTCTTGAGAGAGAGCATC |
| *JcSOC1* | XM_012228124 | qRT-PCR | XK656 F | TTCTTGGACGGCAACGCTTA |
|  |  |  | XK657 R | CTCTCGGAAAAGTGTGGGATC |
| *JcActin1* | NM_112764 | qRT-PCR | XK191 F | CTCCTCTCAACCCCAAAGCCAA |
|  |  |  | XK192 R | CACCAGAATCCAGCACGATACCA |
| *JcDGAT1* | NM_001305997 | qRT-PCR | XD162 F | GCTCTTTGCTTGTATCGTGTG |
|  |  |  | XD163 R | TGGCTGGTAACATAGCGTAG |
| *JcDGAT2* | NM_001306044 | qRT-PCR | XD166 F | GCTATGGAGATGGGCAAACC |
|  |  |  | XD167 R | AAAAGACAATCGGGGTGAACT |
| *JcOLEOSIN* | JQ806305 | qRT-PCR | XE449 F | GGAGCAGCGTTCTTGATATT |
|  |  |  | XE450 R | CACTGGCTACCAAAACTACC |
| *JcWRI1* | NM_001306018 | qRT-PCR | XE447 F | CTACTACCACCAGTCCAAGC |
|  |  |  | XE448 R | AGCCCCCAAATAAACTTGCC |
| caMV35S |  | PCR | XT126 F | CACTATCCTTCGCAAGACCC |
| AtSPL3 | NM_128940 | qRT-PCR | XA752 F | TGAAGGAGATGGAAGATGAGGA |
|  |  |  | XA753 R | AGAAACTGGCGGCATTGAAC |
| AtSPL9 | NM_129782 | qRT-PCR | XA754 F | CAGATACCAAGGTGCCAAGTG |
|  |  |  | XA755 R | GCCACAGTGACTTTAGGTGTTTTA |
| AtSPL15 | LUHQ01000003 | qRT-PCR | XA756 F | AAGTGGAAGGTTGTAGAATGGA |
|  |  |  | XA757 R | TTCTGCGACAACTTCTTTTCTC |
| AtActin2 | NM_112764 | qRT-PCR | XK718 F | TGTGCCAATCTACGAGGGTTT |
|  |  |  | XK719 R | TTTCCCGCTCTGCTGTTGT |
